# Subtypes and Mechanisms of Hypertrophic Cardiomyopathy Proposed by Machine Learning Algorithms

**DOI:** 10.3390/life12101566

**Published:** 2022-10-09

**Authors:** Mila Glavaški, Andrej Preveden, Đorđe Jakovljević, Nenad Filipović, Lazar Velicki

**Affiliations:** 1 Faculty of Medicine, University of Novi Sad, 21000 Novi Sad, Serbia; 2 Institute of Cardiovascular Diseases Vojvodina, 21204 Sremska Kamenica, Serbia; 3 Cardiovascular Research, Translational and Clinical Research and Biosciences Institute, Medicine, Newcastle University, Newcastle upon Tyne Hospitals NHS Foundation Trust, Newcastle upon Tyne NE2 4HH, UK; 4 Cardiovascular and Lifestyle Medicine Theme, Faculty Research Centre (CSELS), Institute for Health and Wellbeing, Faculty of Health Studies, Coventry University, University Hospital Coventry and Warwickshire, Coventry CV1 2TU, UK; 5 Bioengineering Research and Development Center, BioIRC, 34000 Kragujevac, Serbia; 6 Faculty of Engineering, University of Kragujevac, 34000 Kragujevac, Serbia

**Keywords:** hypertrophic cardiomyopathy, machine learning, disease subtypes, disease mechanisms, computational biomedical research

## Abstract

Hypertrophic cardiomyopathy (HCM) is a relatively common inherited cardiac disease that results in left ventricular hypertrophy. Machine learning uses algorithms to study patterns in data and develop models able to make predictions. The aim of this study is to identify HCM subtypes and examine the mechanisms of HCM using machine learning algorithms. Clinical and laboratory findings of 143 adult patients with a confirmed diagnosis of nonobstructive HCM are analyzed; HCM subtypes are determined by clustering, while the presence of different HCM features is predicted in classification machine learning tasks. Four clusters are determined as the optimal number of clusters for this dataset. Models that can predict the presence of particular HCM features from other genotypic and phenotypic information are generated, and subsets of features sufficient to predict the presence of other features of HCM are determined. This research proposes four subtypes of HCM assessed by machine learning algorithms and based on the overall phenotypic expression of the participants of the study. The identified subsets of features sufficient to determine the presence of particular HCM aspects could provide deeper insights into the mechanisms of HCM.

## 1. Introduction

Hypertrophic cardiomyopathy (HCM) is an inherited cardiac disease that results in left ventricular hypertrophy [1]. HCM is relatively common [2], with an incidence of 1 in 500 and some studies suggesting an even higher incidence of 1 in 200 [1,2,3,4]. Symptoms, signs, clinical presentation, and prognosis of HCM are highly heterogeneous and complex [2,4,5,6].

While HCM usually has a stable course with little or no symptomatology [7], it is accountable for significant morbidity and mortality in patients of all ages [3] and is the most common cause of sudden death in the young [2]. Other adverse HCM scenarios are atrial fibrillation (AF) [7] and heart failure (HF) [5,7]. However, the definite subtypes of HCM are not yet identified, and the precise genotype–phenotype associations of HCM [4] and the mechanisms leading to a particular outcome are unknown.

Machine learning uses algorithms to study patterns in data and develop models able to make predictions [8,9]. Cluster analysis is an unsupervised machine learning technique used for finding similar data points in a dataset [10]. Supervised machine learning applies other approaches for making predictions, employing various algorithms [11,12] such as support vector machines [11,12] and random forest [11,12]. Explainable machine learning methods demonstrate the relationships and importance of features used for such predictions [13].

The aim of this study is to identify HCM subtypes and examine the mechanisms of HCM using machine learning algorithms.

## 2. Materials and Methods

### 2.1. Data

Clinical and laboratory findings of 143 adult patients with a confirmed diagnosis of nonobstructive HCM, participants of the SilicoFCM study (NCT03832660) [14,15], were analyzed.

The diagnosis of HCM was defined as maximal left ventricular wall thickness of ≥15 mm (≥13 mm in patients with positive family history of HCM) in the absence of any other cardiac or systemic disease that could cause LV hypertrophy, in line with the European Society of Cardiology guidelines [14,15,16]. Inclusion and exclusion criteria were as described in the design of the SilicoFCM study [14]. Echocardiography and genetic testing were performed as presented in our previous research [15], while cardiopulmonary exercise testing, electrocardiogram (ECG), and ECG Holter monitoring were obtained as specified in the design of the SilicoFCM study [14].

### 2.2. Data Analysis

Most of the data preparation was conducted using Pandas v. 1.4.3, and the data analysis was mainly performed using Scikit-learn v. 1.1.1.

#### 2.2.1. HCM Subtypes

HCM subtypes were determined by clustering.

Only the first visits of the patients were analyzed. The data from second visits were omitted so as not to interfere with cluster analysis. For clustering, the features were used in their raw form; i.e., no combinations of features were made.

Features with more than 30% missing values were removed from the further analysis, and other missing values were imputed by Scikit-learn KNNImputer (n_neighbors = 12, weights = “uniform”). Numerical features were standardized using Scikit-learn StandardScaler. To minimize other data manipulation, KPrototypes algorithm (for datasets with mixed numerical and categorical values) was used for cluster analysis. Furthermore, the elbow method was used for finding the optimal number of clusters, and the result was confirmed using Kneelocator (https://pypi.org/project/kneed/ (accessed on 24 September 2022)). Cluster features were visualized using Seaborn library v. 0.11.2 (https://seaborn.pydata.org (accessed on 24 September 2022)).

Mean values of continuous variables were compared using ANOVA, whereas categorical variables were compared through the chi-square test, using SPSS v. 28.0.1.1. The statistical significance for all tests was set at the *p* value of <0.001.

To the best of our knowledge, there is currently no technique to directly interpret KPrototypes clustering. Therefore, we used indirect methods to interpret it by later decision tree classification (with classes assigned as determined in the clustering), creation of a dendrogram (a visual representation of the decisions that the model makes to determine the class), and computation of feature importance for the later random forest classification. The dendrogram is a result of later decision tree classification (chosen because decision trees are very intuitively explainable) in which belonging to determined clusters (here representing a class) was predicted based on all the data used for clustering. For the visual representation, sklearn.tree.plot_tree was used. Although decision tree results are easy and intuitive to explain, decision trees are greedy algorithms (making locally optimal choices). For more stable and general feature importance, we also performed afterward random forest classification in which belonging to determined clusters (here representing classes) were predicted based on all the data used for clustering. Feature importances for the random forest classification were provided by the fitted attribute of the random forest algorithm (feature_importances_), and they represent impurity-based importance. They are computed as the mean and standard deviation of accumulation of the impurity decrease within each tree.

#### 2.2.2. Prediction of the Presence of HCM Features

The presence of HCM features was predicted as classification machine learning tasks.

A total of 268 visits were analyzed. For most patients, data were obtained for two visits; however, for a small portion, data were collected only for the first visit (longitudinal data for some patients are missing due to loss to follow-up). Measured features were combined and new, engineered features were used in the analysis. Categorical features were combined by their multiplication, categorical and numerical features were combined by their multiplication, and numerical features were combined by division. Moreover, some custom features were made as a sum or multiplication of features congregated together in a meaningful clinical entity.

Features with more than 30% missing values were removed from the further analysis, and other missing values were imputed by Scikit-learn KNNImputer (n_neighbors = 12, weights = “uniform”). Numerical features were standardized using Scikit-learn StandardScaler, while imputation and standardization were performed as a pipeline and applied separately to training and test data. Train sets consisted of 188 (75.80%) visits. Data obtained for two visits for each patient were both assigned to either train or test set only [17]. The selection of the best features for the models was directed by Scikit-learn SelectKBest (score_func = f_classif), Scikit-learn VarianceThreshold (threshold = 0.02), and domain knowledge.

Default values for the Scikit-learn estimators’ parameters were used, while for logistic regression, class_weight = “balanced” was applied.

Accuracy, precision, recall, F1-score, AUC, and average precision (AP, area under the PR curve) were all used as performance metrics, with fivefold cross-validation applied.

In addition, Shap v. 0.41.0 was used for the interpretation of the models. Global feature importance was estimated as mean absolute Shapley values per feature across the data. It indicates the average impact of each feature on model output.

## 3. Results

### 3.1. HCM Subtypes

Four was determined as the optimal number of clusters for this dataset: cluster 0 (*n* = 55), cluster 1 (*n* = 42), cluster 2 (*n* = 17), and cluster 3 (*n* = 29) (Figure 1 and Supplementary Figures S1–S20).

A statistically significant difference between the clusters was found based on: sex, age, weight, BMI, heart murmur, diastolic blood pressure, HCM in family history, genetic disease as comorbidity, LAV, LAVs, MV maxPG, MV meanPG, MVVTI, PLWD, LVIDd, EFLV, LVOT Vmax, LVOT maxPG (Valsalva maneuver), E/E’, AV maxPG, AV meanPG, AV Vmax, AO, AOvs, AcsAO, TR, TAPSE, RVSP, systolic anterior motion, QRS duration, LDH, serum calcium, albumin, and creatinine (Figure 1 and Supplementary Figures S1–S20).

#### 3.1.1. An Approximate Interpretation of Clustering

An approximate estimation of the contribution of each feature to the final results of clustering is shown in Supplement Figures S21 and S22. Due to the indirectness of the interpretability methods used, these interpretations should be anticipated as approximate (they may differ from the process that had happened in the clustering itself).

Based on non-overlapping intervals of each feature in different clusters, together with dendrogram and feature importance estimated, the most important features distinguishing the four HCM subtypes (clusters) determined are: LDH, AO, AOvs, PLWd, LVOT Vmax, MVmeanPG, MVmaxPG, Peak VE/VCO2, presence of heart murmur, AV maxPG, AscAO, AscAO, HCM in family history, serum albumin, weight, LVOT maxPG, MVVTI, AV meanPG, and RVSP.

#### 3.1.2. Association with Genotype

**Figure 2:**
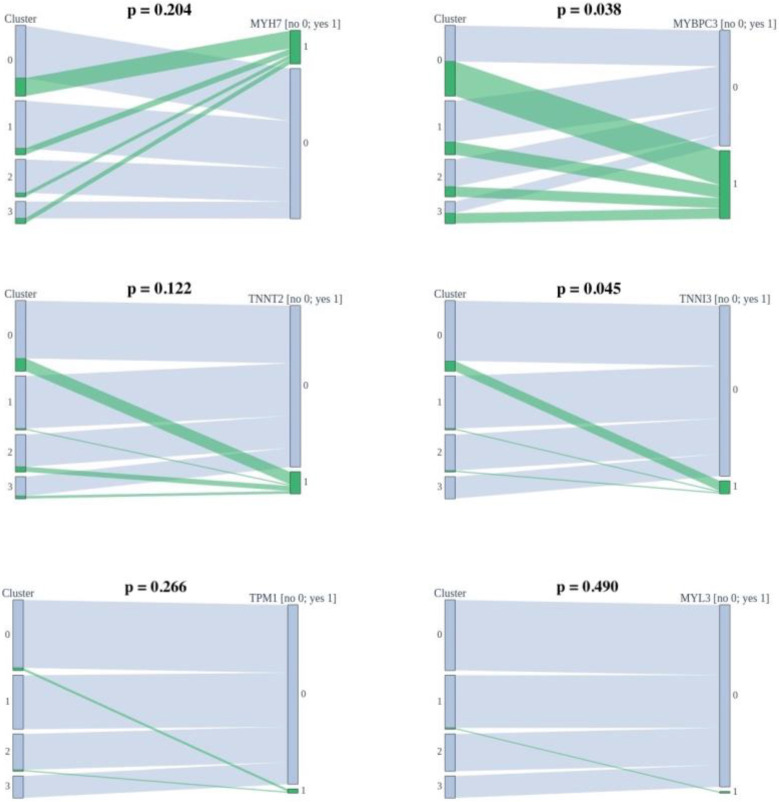
Associations of the determined clusters and found mutations.

Mutations were found in the six causal genes for HCM (*MYH7*, *MYBPC3*, *TNNT2*, *TNNI3*, *TPM1*, and *MYL3*). The associations of the determined clusters and genes in which mutations are found are shown in Figure 2. Clusters do not statistically differ based on the mutated genes.

### 3.2. Prediction of the Presence of HCM Features

Models that can predict the presence of a particular HCM feature from other patient information were generated (Table 1) as described in Section 2.2.2.

Subsets of features sufficient to predict the presence of another HCM aspect by machine learning algorithms were determined (Figure 3, Figure 4, Figure 5, Figure 6, Figure 7, Figure 8, Figure 9, Figure 10, Figure 11, Figure 12, Figure 13, Figure 14, Figure 15, Figure 16, Figure 17, Figure 18, Figure 19, Figure 20, Figure 21, Figure 22 and Figure 23).

#### 3.2.1. Mutated Genes

**Figure 3:**
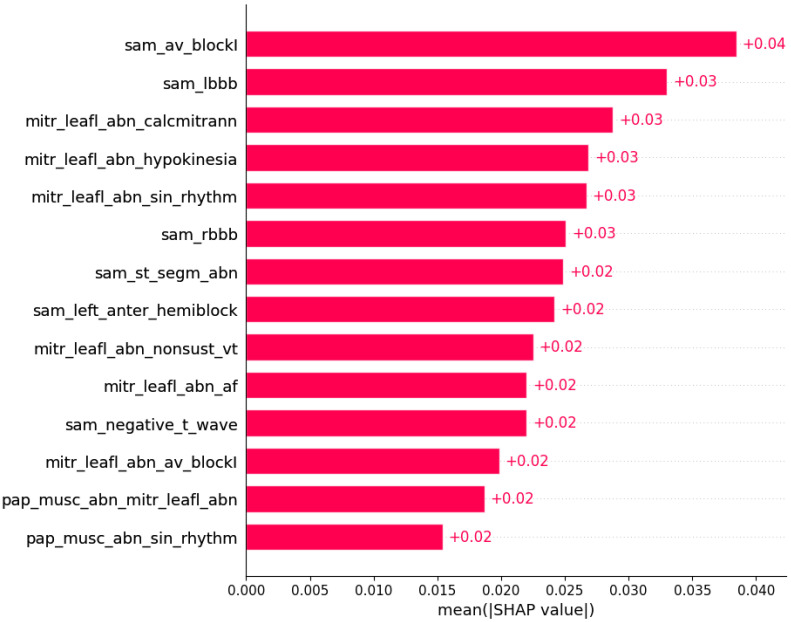
Mutation in the *MYH7* gene was predicted by the shown subset of features. Their relative importance is indicated. (sam_av_blockI = systolic anterior motion x AV block I; sam_lbbb = systolic anterior motion x LBBB; mitr_leafl_abn_calcmitrann = mitral leaflet abnormalities x calcification of mitral annulus; mitr_leafl_abn_hypokinesia = mitral leaflet abnormalities x hypokinesia; mitr_leafl_abn_sin_rhtytm = mitral leaflet abnormalities x sinus rhythm; sam_rbbb = systolic anterior motion x RBBB; sam_st_segm_abn = systolic anterior motion x ST segment abnormalities; sam_left_anter_hemiblock = systolic anterior motion x left anterior hemiblock; mitr_leafl_abn_nonsust_vt = mitral leaflet abnormalities x nonsustained ventricular tachycardia; mitr_leafl_abn_af = mitral leaflet abnormalities x atrial fibrillation; sam_negative_t_wave = systolic anterior motion x negative T wave; mitr_leafl_abn_av_blockI = mitral leaflet abnormalities x AV block I; pap_musc_abn_mitr_leafl_abn = papillary muscle abnormalities x mitral leaflet abnormalities; pap_musc_abn_sin_rhythm = papillary muscle abnormalities x sinus rhythm.)

**Figure 4:**
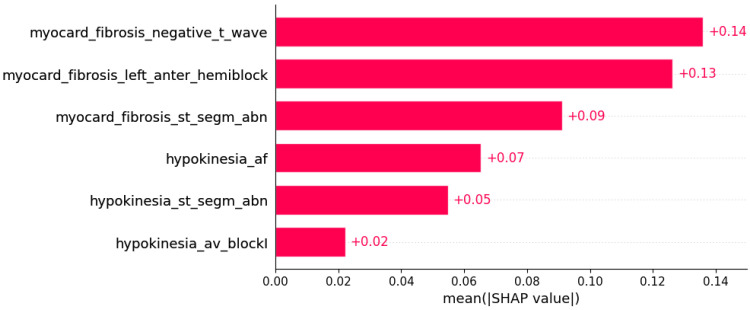
Mutation in the *MYBPC3* gene was predicted by the shown subset of features. Their relative importance is indicated. (myocard_fibrosis_negative_t_wave = myocardial fibrosis x negative T wave; myocard_fibrosis_left_anter_hemiblock = myocardial fibrosis x left anterior hemiblock; myocard_fibrosis_st_segm_abn = myocardial fibrosis x ST segment abnormalities; hypokinesia_af = hypokinesia x atrial fibrillation; hypokinesia_st_segm_abn = hypokinesia x ST segment abnormalities; hypokinesia_av_blockI = hypokinesia x AV block I.)

**Figure 5:**
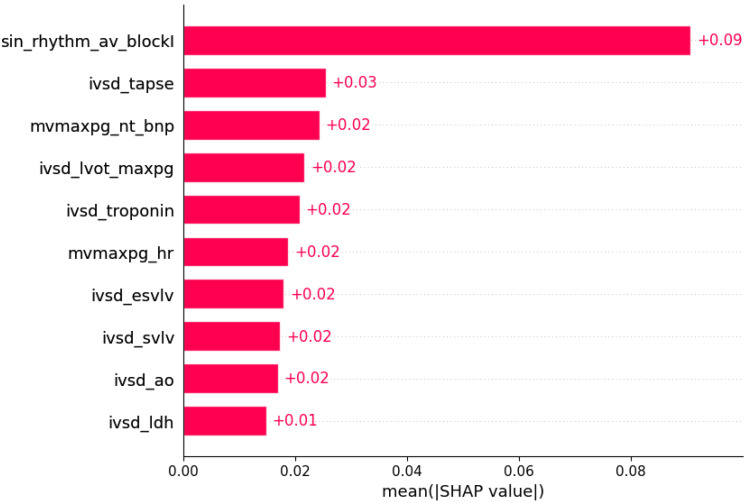
Mutation in the *TNNI3* gene was predicted by the shown subset of features. Their relative importance is indicated. (sin_rhythm_av_blockI = sinus rhythm x AV block I; ivsd_tapse = IVSd/TAPSE; mvmaxpg_nt_bnp = MV maxPG/NT BNP; ivsd_lvot_maxpg = IVSd/LVOT maxPG; ivsd_troponin = IVSd/serum troponin; mvmaxpg_hr = MV maxPG/heart rate; ivsd_esvlv = IVSd/ESVLV; ivsd_svlv = IVSd/SVLV; ivsd_ao = IVSd/AO; ivsd_ldh = IVSd/LDH.)

**Figure 6:**
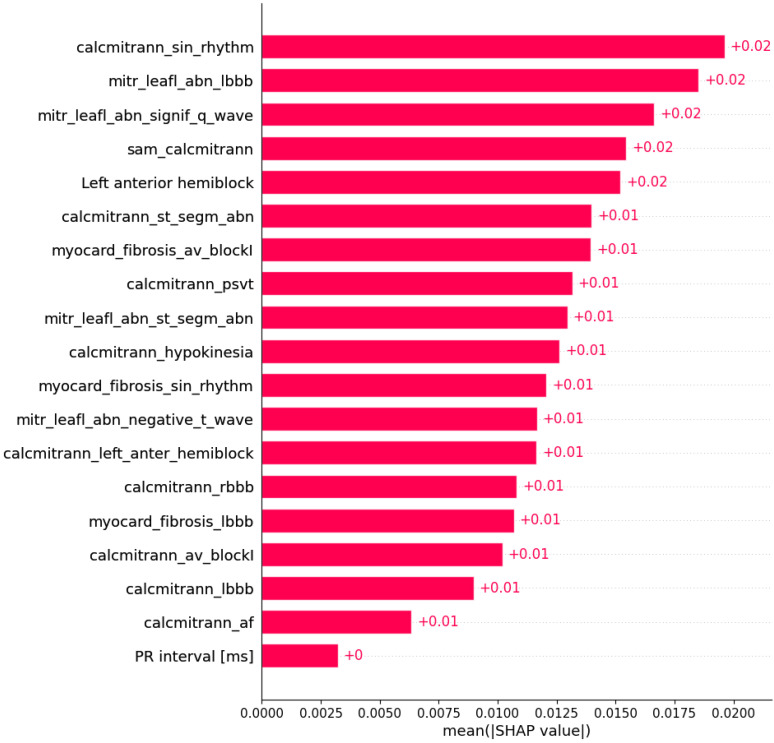
Mutation in the *TNNT2* gene was predicted by the shown subset of features. Their relative importance is indicated. (calcmitrann_sin_rhythm = calcification of mitral annulus x sinus rhythm; mitr_leafl_abn_lbbb = mitral leaflet abnormalities x LBBB; mitr_leafl_abn_signif_q_wave = mitral leaflet abnormalities x significant Q wave; sam_calcmitrann = systolic anterior motion x calcification of mitral annulus; calcmitrann_st_segm_abn = calcification of mitral annulus x ST segment abnormalities; myocard_fibrosis_av_blockI = myocardial fibrosis x AV block I; calcmitrann_psvt = calcification of mitral annulus x paroxysmal supraventricular tachycardia; mitr_leafl_abn_st_segm_abn = mitral leaflet abnormalities x ST segment abnormalities; calcmitrann_hypokinesia = calcification of mitral annulus x hypokinesia; myocard_fibrosis_sin_rhythm = myocardial fibrosis x sinus rhythm; mitr_leafl_abn_negative_t_wave = mitral leaflet abnormalities x negative T wave; calcmitrann_left_anter_hemiblock = calcification of mitral annulus x left anterior hemiblock; calcmitrann_rbbb = calcification of mitral annulus x RBBB; myocard_fibrosis_lbbb = myocardial fibrosis x LBBB; calcmitrann_av_blockI = calcification of mitral annulus x AV block I; calcmitrann_lbbb = calcification of mitral annulus x LBBB; calcmitrann_af = calcification of mitral annulus x atrial fibrillation.)

The presence of mutation in *MYH7*, *MYBPC3*, *TNNI3*, and *TNNT2* genes can be predicted by subsets of phenotypic information (Figure 3, Figure 4, Figure 5 and Figure 6).

#### 3.2.2. Symptoms

**Figure 7:**
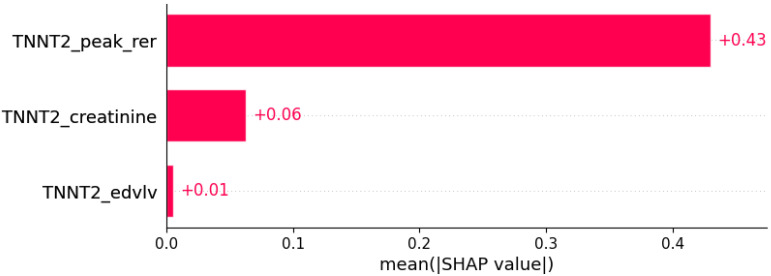
Fatigue was predicted by the shown subset of features. Their relative importance is indicated. (*TNNT2*_peak_rer = mutation in *TNNT2* x peak respiratory exchange ratio; *TNNT2*_creatinine = mutation in *TNNT2* x serum creatinine; *TNNT2*_edvlv = mutation in *TNNT2* x EDVLV.)

**Figure 8:**
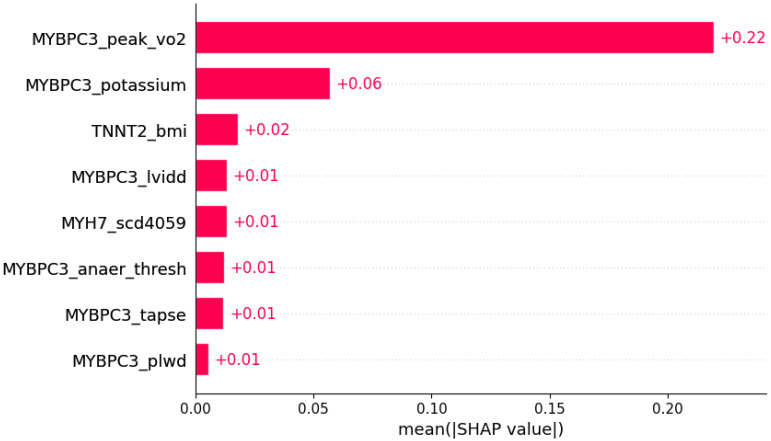
Dyspnea was predicted by the shown subset of features. Their relative importance is indicated. (*MYBPC3*_peak_vo2 = mutation in *MYBPC3* x peak VO2; *MYBPC3*_potassium = mutation in *MYBPC3* x serum potassium; *TNNT2*_bmi = mutation in *TNNT2* x body mass index; *MYBPC3*_lvidd = mutation in *MYBPC3* x LVIDd; *MYH7*_scd4059 = mutation in *MYH7* x SCD in age 40–59 in family history; *MYBPC3*_anaer_thresh = mutation in *MYBPC3* x anaerobic threshold; *MYBPC3*_tapse = mutation in *MYBPC3* x TAPSE; *MYBPC3*_plwd = mutation in *MYBPC3* x PLWD.)

**Figure 9:**
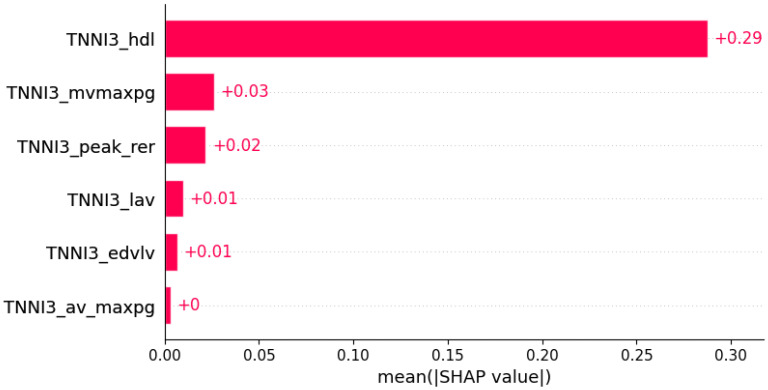
Chest pain was predicted by the shown subset of features. Their relative importance is indicated. (*TNNI3*_hdl = mutation in *TNNI3* x serum HDL; *TNNI3*_mvmaxpg = mutation in *TNNI3* x MV maxPG; *TNNI3*_peak_rer = mutation in *TNNI3* x peak respiratory exchange ratio; *TNNI3*_lav = mutation in *TNNI3* x LAV; *TNNI3*_edvlv = mutation in *TNNI3* x EDVLV; *TNNI3*_av_maxpg = mutation in *TNNI3* x AV maxPG.)

**Figure 10:**
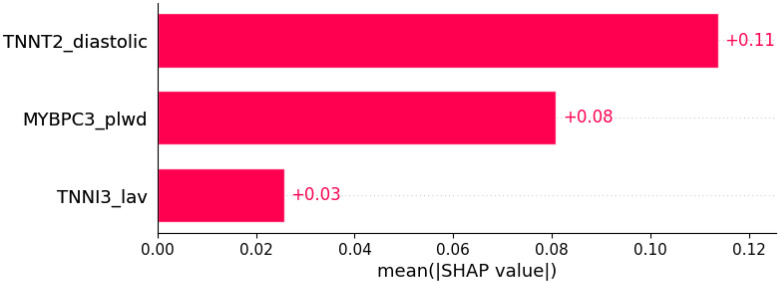
Palpitations were predicted by the shown subset of features. Their relative importance is indicated. (*TNNT2*_diastolic = mutation in *TNNT2* x diastolic blood pressure; *MYBPC3*_plwd = mutation in *MYBPC3* x PLWD; *TNNI3*_lav = mutation in *TNNI3* x LAV.)

**Figure 11:**
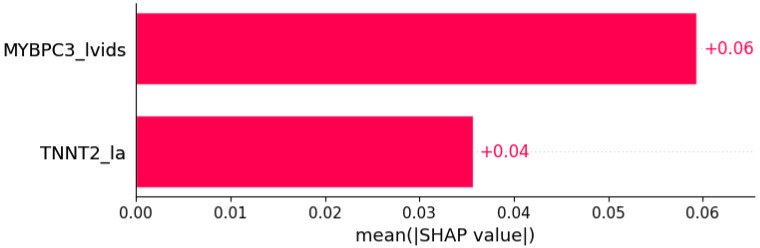
Syncope was predicted by the shown subset of features. Their relative importance is indicated. (*MYBPC3*_lvids = mutation in *MYBPC3* x LVIDs; *TNNT2*_la = mutation in *TNNT2* x LA.)

Symptoms of HCM were predicted by subsets of other genotypic and phenotypic data (Figure 7, Figure 8, Figure 9, Figure 10 and Figure 11).

#### 3.2.3. Signs

**Figure 12:**
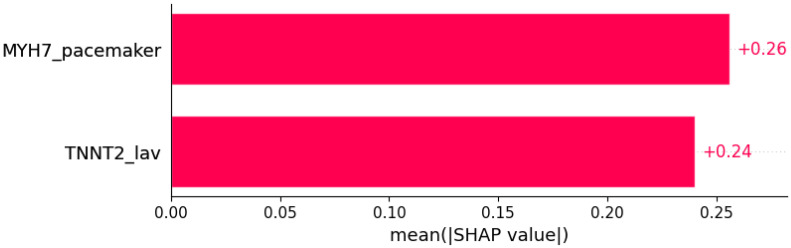
Heart murmur was predicted by the shown subset of features. Their relative importance is indicated. (*MYH7*_pacemaker = mutation in *MYH7* x pacemaker/defibrillator implants in family history; *TNNT2*_lav = mutation in *TNNT2* x LAV.)

**Figure 13:**
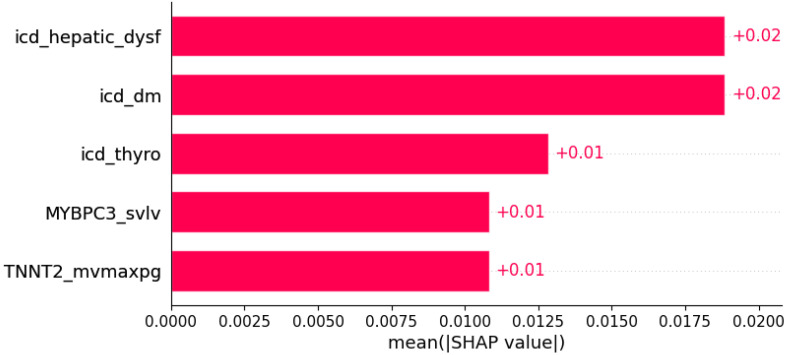
Pretibial edema was predicted by the shown subset of features. Their relative importance is indicated. (icd_hepatic_dysf = ICD x hepatic dysfunction; icd_dm = ICD x diabetes mellitus; icd_thyro = ICD x thyroid disease; *MYBPC3*_svlv = mutation in *MYBPC3* x SVLV; *TNNT2*_mvmaxpg = mutation in *TNNT2* x MVmaxPG;.)

Signs of HCM were predicted by subsets of other genotypic and phenotypic data (Figure 12 and Figure 13).

#### 3.2.4. Echocardiogram

**Figure 14:**
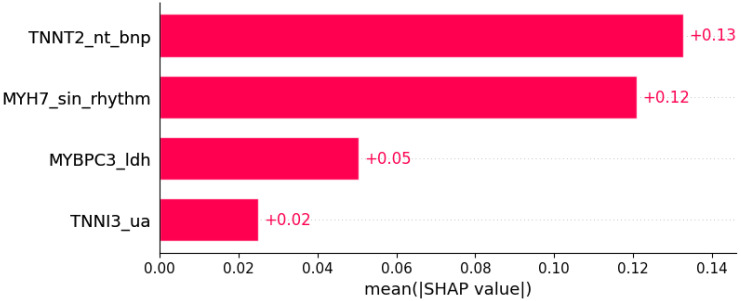
Systolic anterior motion was predicted by the shown subset of features. Their relative importance is indicated. (*TNNT2*_nt_bnp = mutation in *TNNT2* x NT BNP; *MYH7*_sin_rhythm = mutation in *MYH7* x sinus rhythm; *MYBPC3*_ldh = mutation in *MYBPC3* x LDH; *TNNI3*_ua = mutation in *TNNI3* x serum uric acid.)

**Figure 15:**
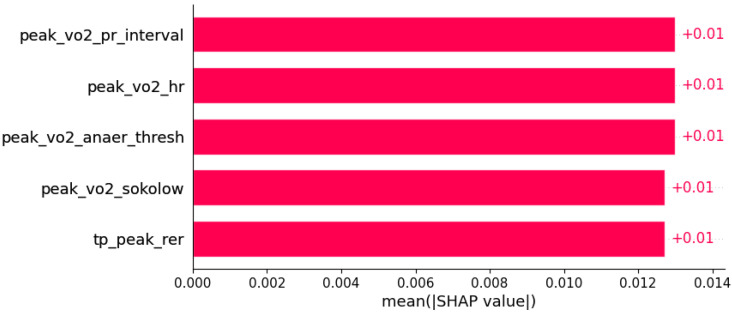
Papillary muscle abnormalities were predicted by the shown subset of features. Their relative importance is indicated. (peak_vo2_pr_interval = peak VO2/PR interval; peak_vo2_hr = peak VO2/heart rate; peak_vo2_anaer_thresh = peak VO2/anaerobic threshold; peak_vo2_sokolow = peak VO2/Sokolow index; tp_peak_rer = total protein in serum/peak respiratory exchange ratio.)

**Figure 16:**
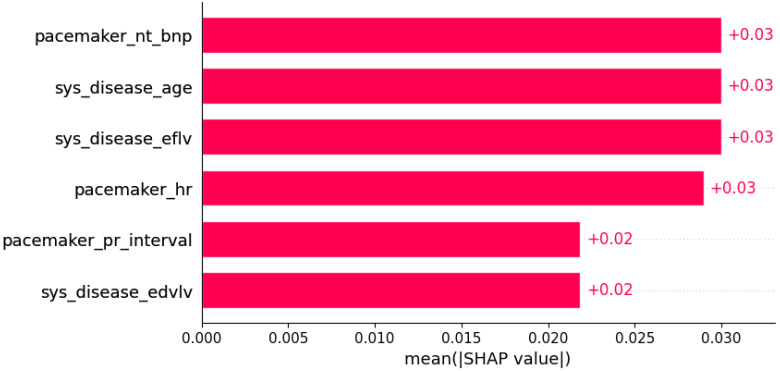
Hypokinesia was predicted by the shown subset of features. Their relative importance is indicated. (pacemaker_nt_bnp = pacemaker/defibrillator implants in family history x NT BNP; sys_disease_age = evidence of systemic disease in family history x age; sys_disease_eflv = evidence of systemic disease in family history x EFLV; pacemaker_hr = pacemaker/defibrillator implants in family history x heart rate; pacemaker_pr_interval = pacemaker/defibrillator implants in family history x PR interval; sys_disease_edvlv = evidence of systemic disease in family history x EDVLV.)

Some ultrasonic findings of HCM were predicted by subsets of other genotypic and phenotypic data (Figure 14, Figure 15 and Figure 16).

#### 3.2.5. Conduction and Rhythm Disorders

**Figure 17:**
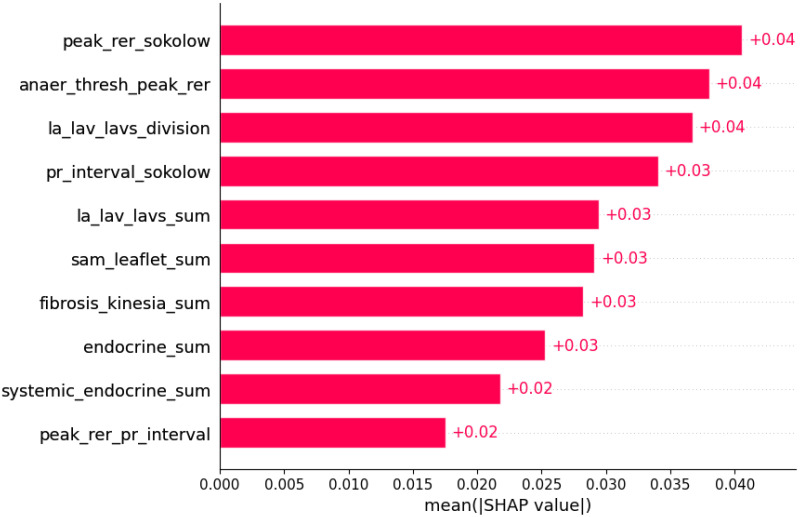
Atrial fibrillation was predicted by the shown subset of features. Their relative importance is indicated. (peak_rer_sokolow = peak respiratory exchange ratio x Sokolow index; anaer_thresh_peak_rer = anaerobic threshold/peak respiratory exchange ratio; la_lav_lavs_division = LA/LAV/LAVs; pr_interval_sokolow = PR interval x Sokolow index; la_lav_lavs_sum = LA+LAV+LAVs; sam_leaflet_sum = systolic anterior motion + mitral regurgitation + papillary muscle abnormalities + mitral leaflet abnormalities + calcification of mitral annulus; fibrosis_kinesia_sum = myocardial fibrosis + hypokinesia + akinesia + dyskinesia + hyperkinesia; endocrine_sum = diabetes mellitus + thyroid disease + phaeochromocytoma + acromegaly; systemic_endocrine_sum = evidence of systemic disease in family history + diabetes mellitus + thyroid disease + phaeochromocytoma + acromegaly + neuromuscular disease + amyloidosis + genetic disease as a comorbidity; peak_rer_pr_interval = peak respiratory exchange ratio/PR interval.)

**Figure 18:**
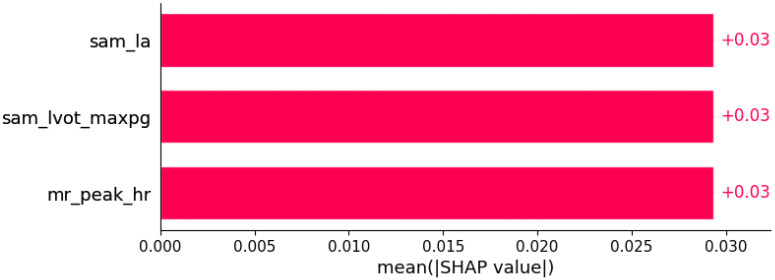
Atrioventricular (AV) block I was predicted by the shown subset of features. Their relative importance is indicated. (sam_la = systolic anterior motion x LA; sam_lvot_maxpg = systolic anterior motion x LVOT maxPG; mr_peak_hr = mitral regurgitation x peak HR in cardiopulmonary exercise test.)

**Figure 19:**
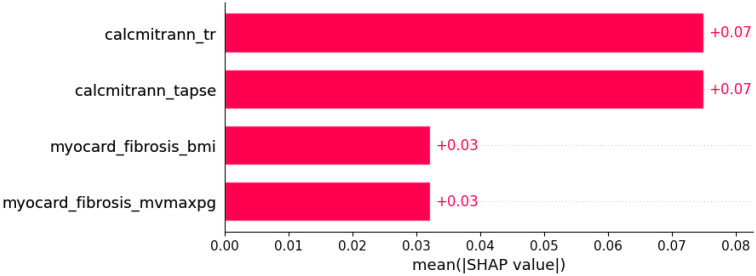
Left bundle branch block (LBBB) was predicted by the shown subset of features. Their relative importance is indicated. (calcmitrann_tr = calcification of mitral annulus x tricuspid regurgitation; calcmitrann_tapse = calcification of mitral annulus x TAPSE; myocard_fibrosis_bmi = myocardial fibrosis x body mass index; myocardial_fibrosis_mvmaxpg = myocardial fibrosis x MV maxPG.)

**Figure 20:**
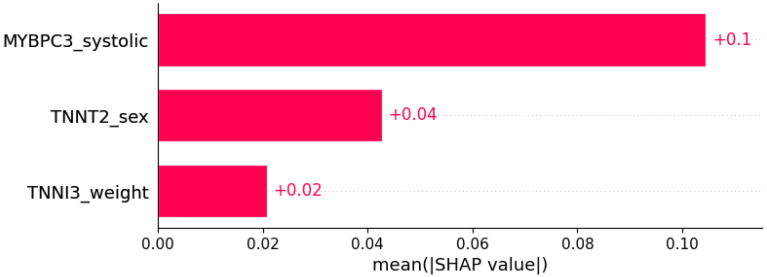
Right bundle branch block (RBBB) was predicted by the shown subset of features. Their relative importance is indicated. (*MYBPC3*_systolic = mutation in *MYBPC3* x systolic blood pressure; *TNNT2*_sex = mutation in *TNNT2* x sex; *TNNI3*_weight = mutation in *TNNI3* x weight.)

**Figure 21:**
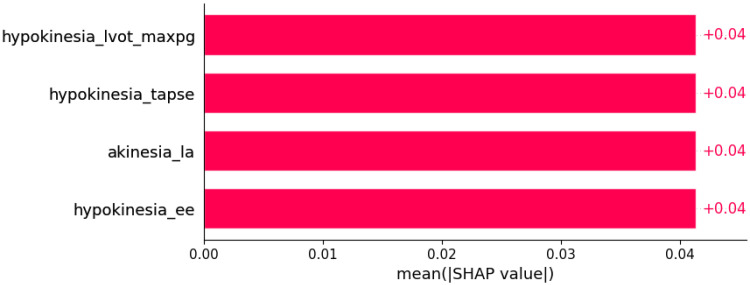
Left anterior hemiblock was predicted by the shown subset of features. Their relative importance is indicated. (hypokinesia_lvot_max_pg = hypokinesia x LVOT maxPG; hypokinesia_tapse = hypokinesia x TAPSE; akinesia_la = akinesia x LA; hypokinesia_ee = hypokinesia x E/E’.)

Some conduction and rhythm disorders in HCM were predicted by subsets of other genotypic and phenotypic data (Figure 17, Figure 18, Figure 19, Figure 20 and Figure 21).

#### 3.2.6. Ischemia

**Figure 22:**
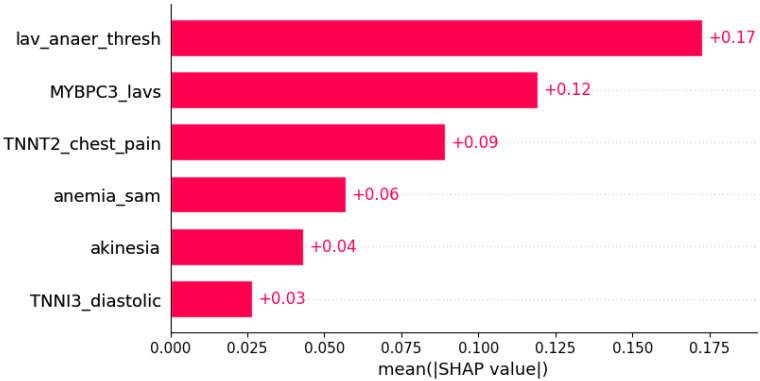
ST segment abnormalities were predicted by the shown subset of features. Their relative importance is indicated. (lav_anaer_thesh = LAV/anaerobic threshold; *MYBPC3*_lavs = mutation in *MYBPC3* x LAVs; *TNNT2*_chest_pain = mutation in *TNNT2* x chest pain; anemia_sam = anemia x systolic anterior motion; *TNNI3*_diastolic = mutation in *TNNI3* x diastolic blood pressure.)

**Figure 23:**
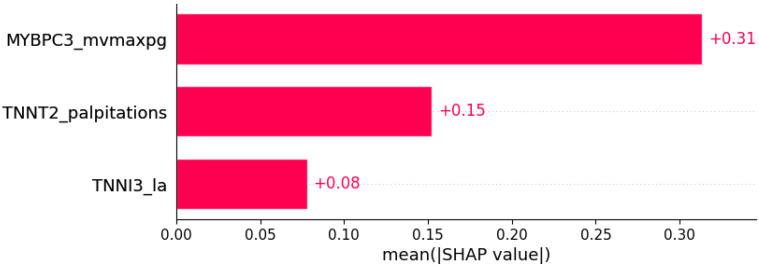
Negative T wave was predicted by the shown subset of features. Their relative importance is indicated. (*MYBPC3*_mvmaxpg = mutation in *MYBPC3* x MV maxPG; *TNNT2*_palpitations = mutation in *TNNT2* x palpitations; *TNNI3*_la = mutation in *TNNI3* x LA.)

ECG findings indicating myocardial ischemia in HCM were predicted by subsets of other genotypic and phenotypic data (Figure 22 and Figure 23).

## 4. Discussion

### 4.1. HCM Subtypes

HCM has usually been divided into subtypes based on the hypertrophy morphology sites (e.g., apical, midventral, and basal) [18].

A study by Tang et al. shows that the prognoses for different morphological types after surgical myectomy are different [18].

Traditionally, there are two types of HCM: the more common obstructive HCM (70% of cases, with left ventricular outflow obstruction) and the less common nonobstructive type (30% of cases) [19]. The American Heart Association Working Group suggests that HCM should be defined genetically [20], while the European Society of Cardiology Working Group recommends morphological classification [21].

In 1981, Maron et al. defined four types of HCM, depending on which part hypertrophy involves: type I: basal septum; type II: whole septum; type III: septum, anterior, and anterolateral walls; type IV: apical LV [22,23].

Syed et al. suggest at least five major anatomic subsets based on the septal contour, location, and extent of hypertrophy: reverse curvature, sigmoidal septum, neutral contour, apical form, and mid-ventricular form [23,24].

Furthermore, Helmy et al. propose a classification based on the different patterns of hypertrophy: pattern 1: septum alone; pattern 2: septum and adjacent segments (not apical segment); pattern 3: apical in combination with other LV segments; and pattern 4: apical [23,25].

Additionally, Parato et al. have shown that echocardiographic patterns have a significant impact on the clinical course and prognosis of HCM [23].

Kim et al. examined differences in apical and non-apical types of HCM and concluded that apical forms are associated with less severe myocardial fibrosis and diastolic dysfunction, and subsequently milder clinical presentation and better prognosis when compared with other forms of HCM [26].

This paper provides the first attempt to define the various types of HCM based on overall phenotypic appearance and represents a step toward HCM precision medicine, which could eventually facilitate the creation of prevention and treatment strategies specifically developed for particular groups of HCM patients.

Although statistically significant differences were found between four HCM subtypes (clusters), for some features, the overlapping intervals of their values hinder their implementation in separating all the subtypes (clusters) from each other.

Our results suggest four HCM subtypes: cluster 0, distinguishable by “AHOLD” (word scrambled from LDH and AO, from whose values the cluster is distinguishable) with values mainly being LDH > 300 U/L, AO > 30 mm, MVmaxPG < 2 mmHg, PLWD < 12 mm, LVOT Vmax < 2 m/s, and serum albumin > 44 g/L; cluster 1, distinguishable by “RVSP ASCAOVS” (RVSP, AscAO, and AOvs) with values mainly being AOvs > 27 m/s, AscAO < 31 mm, PLWD < 12 mm, LVOT Vmax < 2 m/s, RVSP < 28 mmHg, MVmeanPG < 1 mmHg, and serum albumin > 44 g/L; cluster 2, distinguishable by weight with values mainly being weight > 95 kg, PLWD > 12 mm, LVOT Vmax > 2 m/s, and serum albumin < 43 g/L; and cluster 3, distinguishable by “AV LVOT PG” (AV meanPG, AV maxPG, and LVOT maxPG) with values mostly being LVOT maxPG > 15 mmHg, LVOT Vmax > 2 m/s, MVmeanPG > 2 mmHg, MVmaxPG > 5 mmHg, PLWD > 12 mm, AV meanPG > 6 mmHg, AV maxPG > 15 mmHg, and serum albumin < 43 g/L.

Cluster 3 mainly consists of women, the patients in clusters 2 and 3 are older than those in clusters 0 and 1, and in cluster 2, the patients are more obese. Moreover, in clusters 0 and 3, heart murmur is present in most cases, while it represents a rarity in clusters 1 and 2. Diastolic blood pressure is the highest in cluster 0. HCM in family history is most often present in the majority of cases in cluster 1. Genetic disease as comorbidity is most often present in cluster 0. Systolic anterior motion is most often present in cluster 3 and absent in cluster 1. LDH is the highest in cluster 0, while creatinine is higher in cluster 2.

Echocardiography parameters showed that cluster 3 had the smallest diameters and volumes of the left ventricle in both systole and diastole with the highest thickness of the interventricular septum, while cluster 2 had the highest measurements of the left ventricle cavity. Accordingly, left ventricular systolic function expressed through ejection fraction was greatest in cluster 3 and lowest in cluster 2. However, cluster 3 had the most impaired diastolic function and the highest left ventricular filling pressures expressed through E/e’.

Cluster 0 could be described as consisting of younger patients with heart murmur, higher diastolic blood pressure, and higher LDH values.

Cluster 1 is also made up of younger patients, usually without heart murmur and systolic anterior motion, but with HCM in their family history.

Cluster 2 involves older, more obese males, usually without heart murmur and with relatively higher serum creatinine. It has the highest measurements of the left ventricle cavity and the lowest left ventricular systolic function.

Cluster 3 is mainly a female cluster, consisting of older patients, usually with heart murmur and systolic anterior motion in around 60% of cases. It has the smallest diameters and volumes of the left ventricle and the highest thickness of the interventricular septum, as well as the greatest left ventricular systolic function. In addition, it has the most impaired diastolic function and the highest left ventricular filling pressures.

### 4.2. Prediction of the Presence of HCM Features

Some of associations shown in Figure 3, Figure 4, Figure 5, Figure 6, Figure 7, Figure 8, Figure 9, Figure 10, Figure 11, Figure 12, Figure 13, Figure 14, Figure 15, Figure 16, Figure 17, Figure 18, Figure 19, Figure 20, Figure 21, Figure 22 and Figure 23 are already described in the literature: The presence of systolic anterior motion and mitral leaflet abnormalities was shown to be more frequent in patients with mutation in the *MYH7* gene, and calcifications of mitral annulus were registered only in *MYH7* patients [27]. A higher degree of mitral valve regurgitation is found in patients with a mutation in the *MYH7* gene [28], and *MYH7* is proposed as one of the genes that are most commonly mutated in early-onset AF [29]; HCM patients with likely pathogenic or pathogenic mutations in *MYH7* had a higher rate of incident AF compared with other sarcomeric genes [30]. AF was found to be independently associated with *MYH7* variants amongst sarcomere-positive HCM [31], and a higher frequency of AF was found in patients with mutation in the *MYH7* gene [27,32]. Missense mutations in *MYBPC3* gene are proposed to be responsible for AV block [33].

Dyspnea has been reported as a factor associated with left ventricular dilatation in hypertrophic cardiomyopathy [34], while higher prevalence rates of moderate to severe dyspnea were found in hypertensive patients with reduced TAPSE [35]. In hepatic and thyroid disorders as well as diabetes mellitus, pretibial edema might be found [36,37,38,39].

A higher anaerobic threshold in HF patients with AF is reported compared with HF patients with sinus rhythm [40]. Left atrial (LA) remodeling represents an important substrate for AF [41], and AF is associated with LA enlargement [42].

PR interval might be considered a predictor for AF, with both high and low extremes associated with AF risk [43]. PR interval prolongation and AF share similar characteristics, and PR interval prolongation has been proposed as a possible preliminary stage for AF [44]. AF is suggested to be of key importance in the development of AF in HF [45]. LA fibrosis is an important event in AF pathogenesis and a risk factor for adverse outcomes in AF [46]. LA dyskinesia observed in the LASct4c and 4c views are proposed as independent risk factors for AF recurrence following direct current cardioversion [47]. Endocrine factors play an important role in AF pathogenesis, and endocrine dysfunction promotes AF [48]. Mitral regurgitation is sometimes associated with AV block [49,50,51]. RBBB is more frequent in men [52,53,54,55].

Ischemia is associated with a shorter time to anaerobic threshold in HCM patients [56].

### 4.3. Technical and Statistical Aspects

The categorical features as part of combinations in this study have a “stop or pass the value of the other feature” effect: when a categorical feature is negative (0), the multiplication with another feature will produce 0 and nullify the value of another feature; however, when it is positive (1), the other feature will retain its value.

Although the influence of a single feature could generally be both positive and negative, all combinations of features and predicted outcomes shown in this research are directly correlated (with predicted feature positive if the shown combinations of features are positive or larger, and vice versa). While we observed such trends in SHAP waterfall plots that were inspected on a case-to-case basis, these findings are too extensive to be presented in this paper.

In the feature selection phase, we excluded all feature combinations that appeared completely clinically illogical.

Sets of features sufficient to “predict” a particular outcome should not be perceived as predictors, but rather as a mixture of associations, causations, and co-expressions with the “predicted” features. Some of these are already known, and more might be indicated in rare studies; nevertheless, there are some that are completely unknown.

### 4.4. Limitations

“Cut-off” values for separating the clusters are estimated for the analyzed dataset and need further confirmation or refinement before they could be used as cut-offs of any kind.

In the prediction of the presence of HCM features, the models’ performance appears to be unexpectedly good. However, we performed an additional analysis to check the overall approach. For each of the presented predictions, we created models using different machine learning algorithms: decision tree, random forest, logistic regression, ridge classifier, linear SVC, and RBF kernel SVC. In this paper, we presented only the best results. However, some of the non-shown models had performance metrics around 0.75. Since feature importance in general is more reliable for good classifiers than for moderate ones, and since the focus of this study was on features that might indicate the presence of another feature, we proceeded with models with better performance. The completely same methodology was applied for the classification of the presence of the same features based on the genetic data only (presence of mutations in different genes), and models produced had AUC values around or below 0.5. Although there were many different genetic features, both in raw and engineered form (following the same rules for combining them as presented in this manuscript), the performance was no better than random guessing. To verify the methodology and check if some kind of feature-overfitting might produce such a good performance, we created an artificial feature with the value of 0 for all even-index patients and the value of 1 for all odd-index patients in the database (patients in the database were already randomly ordered). We applied the same methodology and again obtained AUC values around or below 0.5. Furthermore, in choosing the features to be included in predictions of other features, we removed all with a high number of null values, to exclude the possibility that the model learns a few of them “by heart” and combines them to produce a good result (i.e., learns the peculiarities of the dataset). We also removed all the features that might provide direct answers to the models (for the questions asked in classifications), to exclude the possibility of data leakage. In the end, a possible explanation for these results could be that this is probably a trivial computational task, with some of these features (or them altogether) immediately giving the correct answers to the models (whether or not “predicted” features will be clinically presented). However, not all of them are yet known as direct cause-and-effect or very-common-association combinations, especially since we are here dealing with the combinations of features (rarely examined as such in classical clinical research). Despite all the actions taken to overcome possible reasons for such perfect performance, these results must be taken critically and observed as possibilities that need further confirmation.

We do not claim that shown features in the prediction of the presence of other HCM features are the best to predict shown outcomes; they were sufficient to predict the shown features for this dataset, after removal of all the features that might mislead models and dis-reflect actual associations and relationships.

Presented results reflect statistical distributions contained in the data analyzed and need further confirmation in other similar datasets or further investigation in clinical settings.

In general, the machine learning algorithms used in this research are utilized to predict important features based on other genotypic and phenotypic information. Some features predicted in this paper would not be interesting or useful in clinical practice. However, the sets and combinations of features that are sufficient to “predict” the features shown might reveal some unknown associations between clinical presentations.

## 5. Conclusions

This research has proposed four subtypes of HCM assessed by machine learning algorithms and based on the overall phenotype expressed by the participants of the study. The most important features distinguishing the four HCM subtypes determined are: LDH, AO, AOvs, PLWd, LVOT Vmax, MVmeanPG, MVmaxPG, Peak VE/VCO2, presence of heart murmur, AV maxPG, AscAO, AscAO, HCM in family history, serum albumin, weight, LVOT maxPG, MVVTI, AV meanPG, and RVSP. This could represent a step toward HCM precision medicine. In addition, subsets of features sufficient to determine the presence of particular HCM features from other genotypic and phenotypic information by machine learning algorithms are determined; these could provide deeper insights into the mechanisms of HCM.

## Figures and Tables

**Figure 1:**
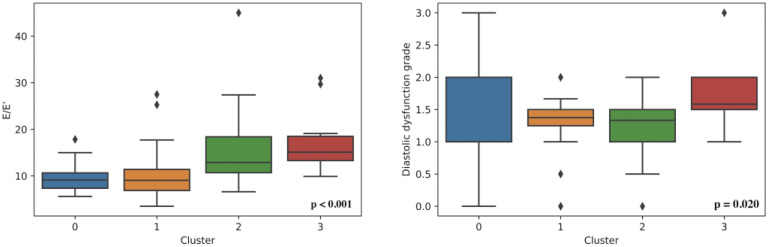
Left ventricle diastolic dysfunction in determined clusters.

**Table 1:** Performance of generated models.

Feature Predicted	Estimator	Accuracy	Precision	Recall	F1-Score	AUC from Estimator	AUC from Predictions	PR from Estimator	PR from Predictions
Mutation in *MYH7*	SVC	1.00	1.00	1.00	1.00	1.00	1.00	1.00	1.00
Mutation in *MYBPC3*	SVC	1.00	1.00	1.00	1.00	1.00	1.00	1.00	1.00
Mutation in *TNNI3*	SVC	1.00	1.00	1.00	1.00	1.00	1.00	1.00	1.00
Mutation in *TNNT2*	SVC	1.00	1.00	1.00	1.00	1.00	1.00	1.00	1.00
Fatigue	SVC	1.00	1.00	1.00	1.00	1.00	1.00	1.00	1.00
Dyspnea	SVC	1.00	1.00	1.00	1.00	1.00	1.00	1.00	1.00
Chest pain	RF	1.00	1.00	1.00	1.00	1.00	1.00	1.00	1.00
Palpitations	SVC	0.97	0.97	0.83	0.91	0.92	0.92	0.86	0.86
Syncope	SVC	1.00	1.00	1.00	1.00	1.00	1.00	1.00	1.00
Heart murmur	SVC	0.89	0.82	0.76	0.87	0.88	0.88	0.88	0.88
Pretibial edema	SVC	1.00	1.00	1.00	1.00	1.00	1.00	1.00	1.00
Systolic anterior motion	SVC	0.99	0.98	0.93	0.97	0.97	0.97	0.95	0.95
Papillary muscle abnormalities	LogReg	1.00	1.00	1.00	1.00	1.00	1.00	1.00	1.00
Hypokinesia	SVC	1.00	1.00	1.00	1.00	1.00	1.00	1.00	1.00
Atrial fibrillation	SVC	1.00	1.00	1.00	1.00	1.00	1.00	1.00	1.00
AV block I	SVC	1.00	1.00	1.00	1.00	1.00	1.00	1.00	1.00
LBBB	SVC	1.00	1.00	1.00	1.00	1.00	1.00	1.00	1.00
RBBB	SVC	1.00	1.00	1.00	1.00	1.00	1.00	1.00	1.00
Left anterior hemiblock	SVC	1.00	1.00	1.00	1.00	1.00	1.00	1.00	1.00
ST segment abnormalities	RF	0.93	0.81	0.89	0.90	0.97	0.94	0.98	0.96
Negative T wave	SVC	0.93	0.88	0.85	0.92	0.93	0.93	0.92	0.92

SVC: radial basis function (RBF) kernel C-support vector classification; RF: random forest; LogReg: logistic regression.

## Data Availability

Data available on reasonable request.

## Supplementary Materials

The following supporting information can be downloaded at: https://www.mdpi.com/article/10.3390/life12101566/s1, Figure S1: Sex; Figure S2: Age, weight, and BMI; Figure S3: Symptoms and signs; Figure S4: Blood pressure; Figure S5: Family history; Figure S6: Comorbidities; Figure S7: Left atrial size; Figure S8: Pressure gradients across mitral valve; Figure S9: Left ventricle; Figure S10: Left ventricle outflow tract pressure gradients; Figure S11: Left ventricle wall motion abnormalities; Figure S12: Aortic valve and proximal aorta; Figure S13: Right ventricle and tricuspid valve; Figure S14: Mitral apparatus irregularities, mitral regurgitation, and systolic anterior motion; Figure S15: CPET; Figure S16: Rhythm and conduction; Figure S17: Ischemia and Sokolow index; Figure S18: Enzymes and cardiac biomarkers; Figure S19: General clinical chemistry; Figure S20: Lipid metabolism; Figure S21: An approximate interpretation of clustering—dendrogram; Table S1: An approximate interpretation of clustering—feature importance.
