# Supplementary material for: Subtypes and Mechanisms of Hypertrophic Cardiomyopathy Proposed by Machine Learning Algorithms
Source: Life (Basel). 2022 Oct 9;12(10):1566. doi: 10.3390/life12101566 (PMC9605444; doi:10.3390/life12101566)
Supplement: Supplementary file 1 [file life-12-01566-s001.zip › Tabel S1, An approximate interpretation of clusteringí¬feature importance.pdf]

Table S1. An approximate interpretation of clustering—feature importance

Feature	Feature importance
LDH	0.072933
AO	0.063513
AOvs	0.053407
PLWd	0.042644
LVOT Vmax	0.039411
MVmeanPG	0.034072
MVmaxPG	0.032063
Peak VE/VCO2	0.031522
Heart murmur	0.027068
AV maxPG	0.026379
AscAO	0.024348
HCM*	0.024280
Albumin	0.023722
Weight	0.017189
LVOT maxPG	0.017161
MVVTI	0.015820
AV meanPG	0.014308
RVSP	0.014291
NYHA class	0.013766
AV Vmax	0.013508
LA	0.013420
AVVTI	0.013285
BMI	0.013265
LAV	0.013225
Diastolic	0.013080
LAVs	0.012232
QRS duration	0.011427
LVIDd	0.011076
Calcium	0.010857
NT-BNP	0.009222
AVA by planimetry	0.009159

E/E'	0.009069
RAVs	0.008986
EFLV	0.008804
Creatinine	0.008517
LVOT maxPG (Valsalva maneuver)	0.008415
Heart rate	0.008279
LDL	0.007798
Age	0.007743
IVSd	0.007711
Sokolow index	0.007636
MR	0.007281
Peak HR	0.006946
Anaerobic threshold	0.006793
ESVLV	0.006754

*In family history
