# Supplementary figures and images for: Subtypes and Mechanisms of Hypertrophic Cardiomyopathy Proposed by Machine Learning Algorithms
Source: Life (Basel). 2022 Oct 9;12(10):1566. doi: 10.3390/life12101566 (PMC9605444; doi:10.3390/life12101566)

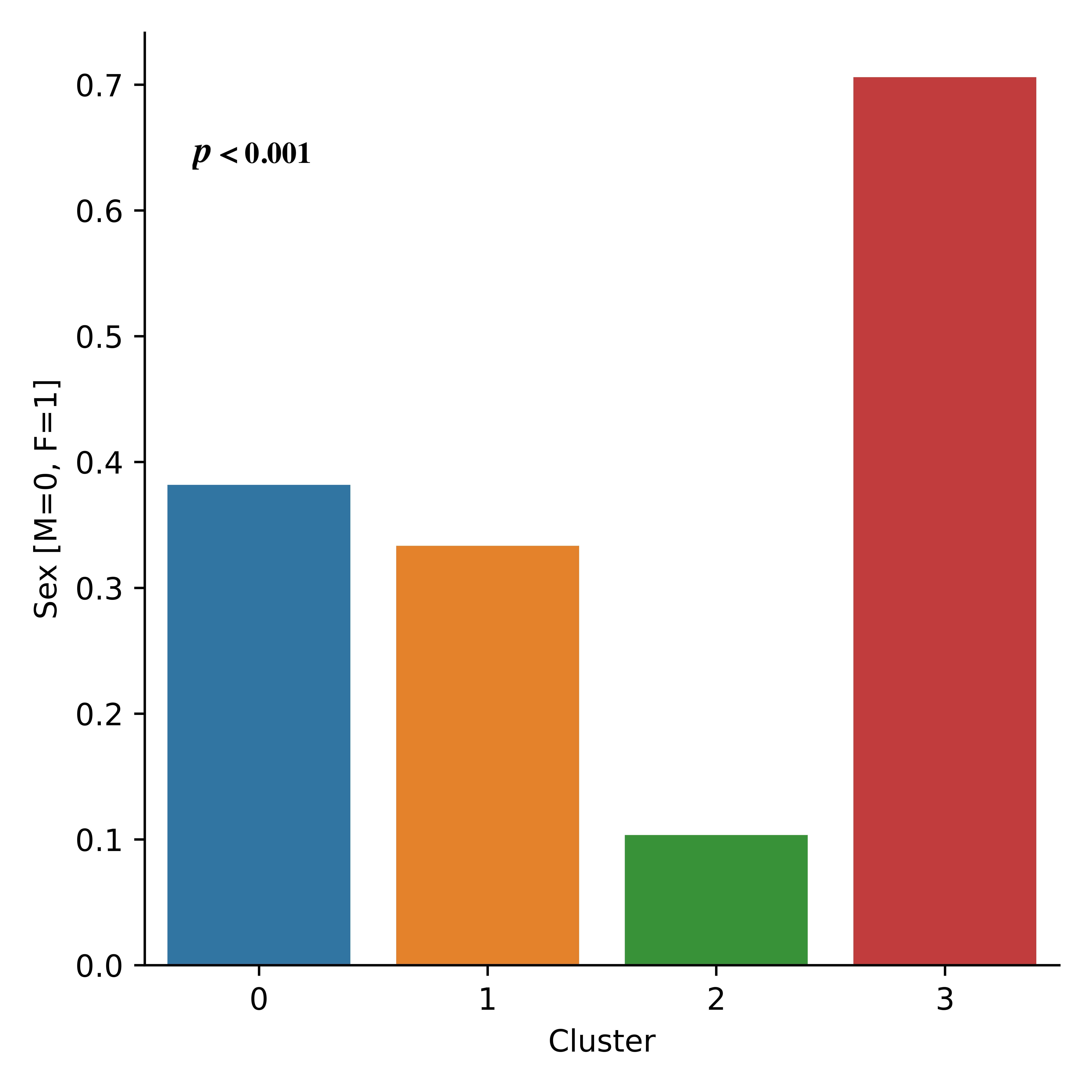

Supplement: Supplementary file 1 [file life-12-01566-s001.zip › Figure S1, Sex.png]

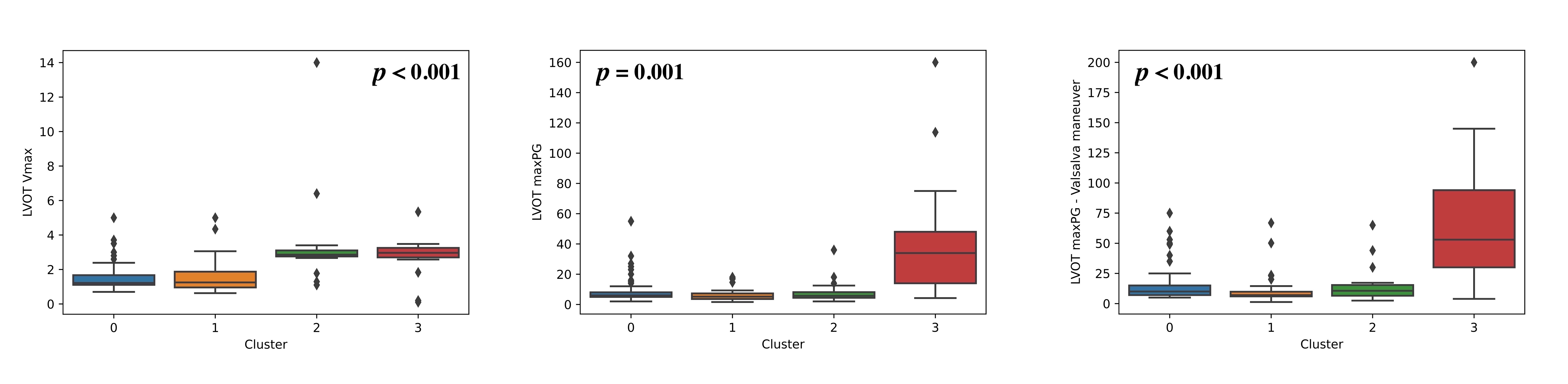

Supplement: Supplementary file 1 [file life-12-01566-s001.zip › Figure S10, Left ventricle outflow tract pressure gradients.jpg]

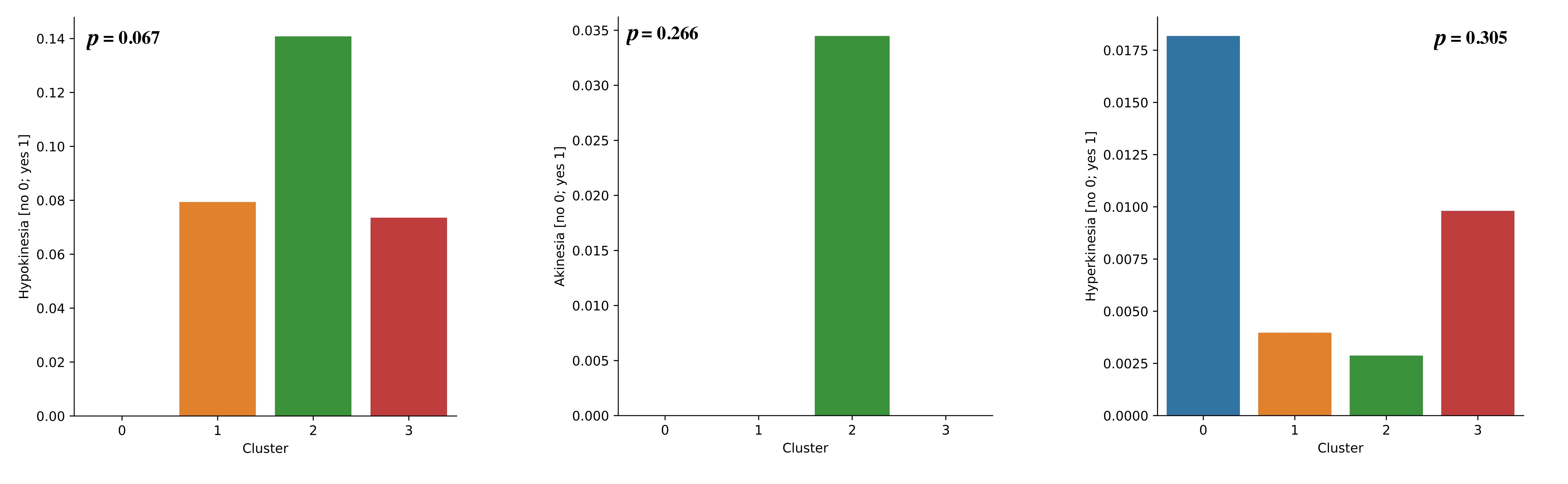

Supplement: Supplementary file 1 [file life-12-01566-s001.zip › Figure S11, Left ventricle wall motion abnormalities.jpg]

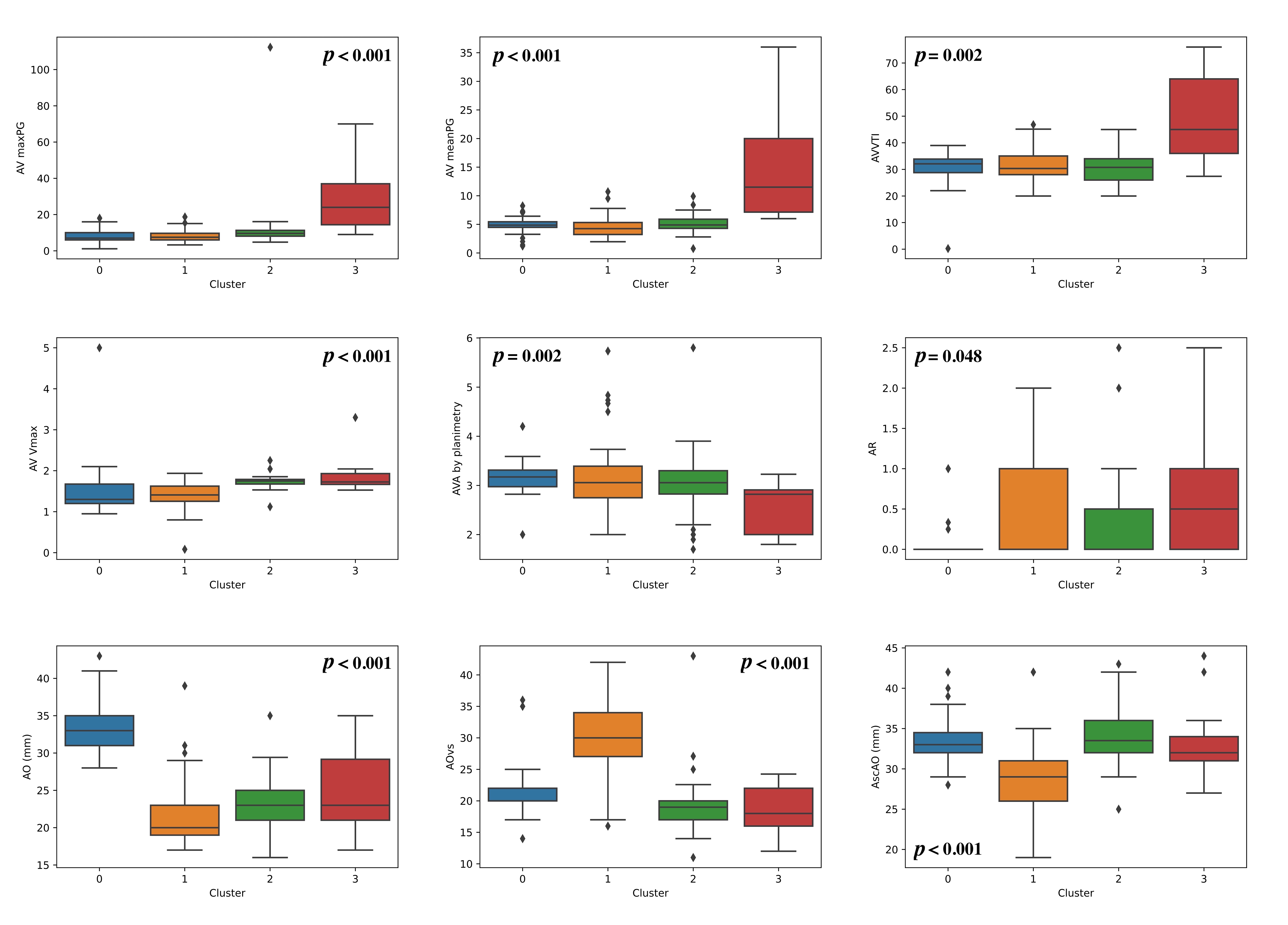

Supplement: Supplementary file 1 [file life-12-01566-s001.zip › Figure S12, Aortic valve and proximal aorta.jpg]

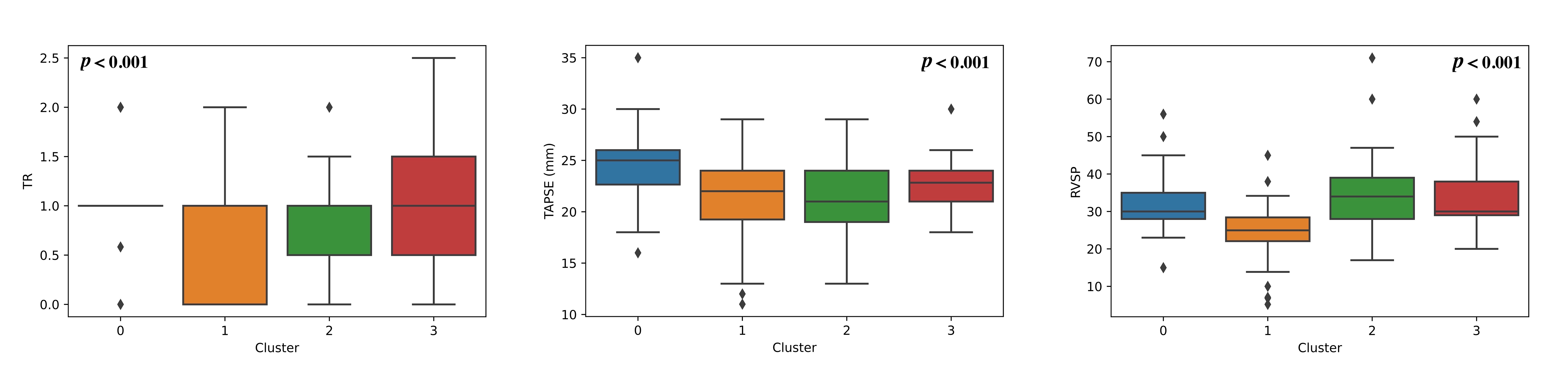

Supplement: Supplementary file 1 [file life-12-01566-s001.zip › Figure S13, Right ventricle and tricuspid valve.jpg]

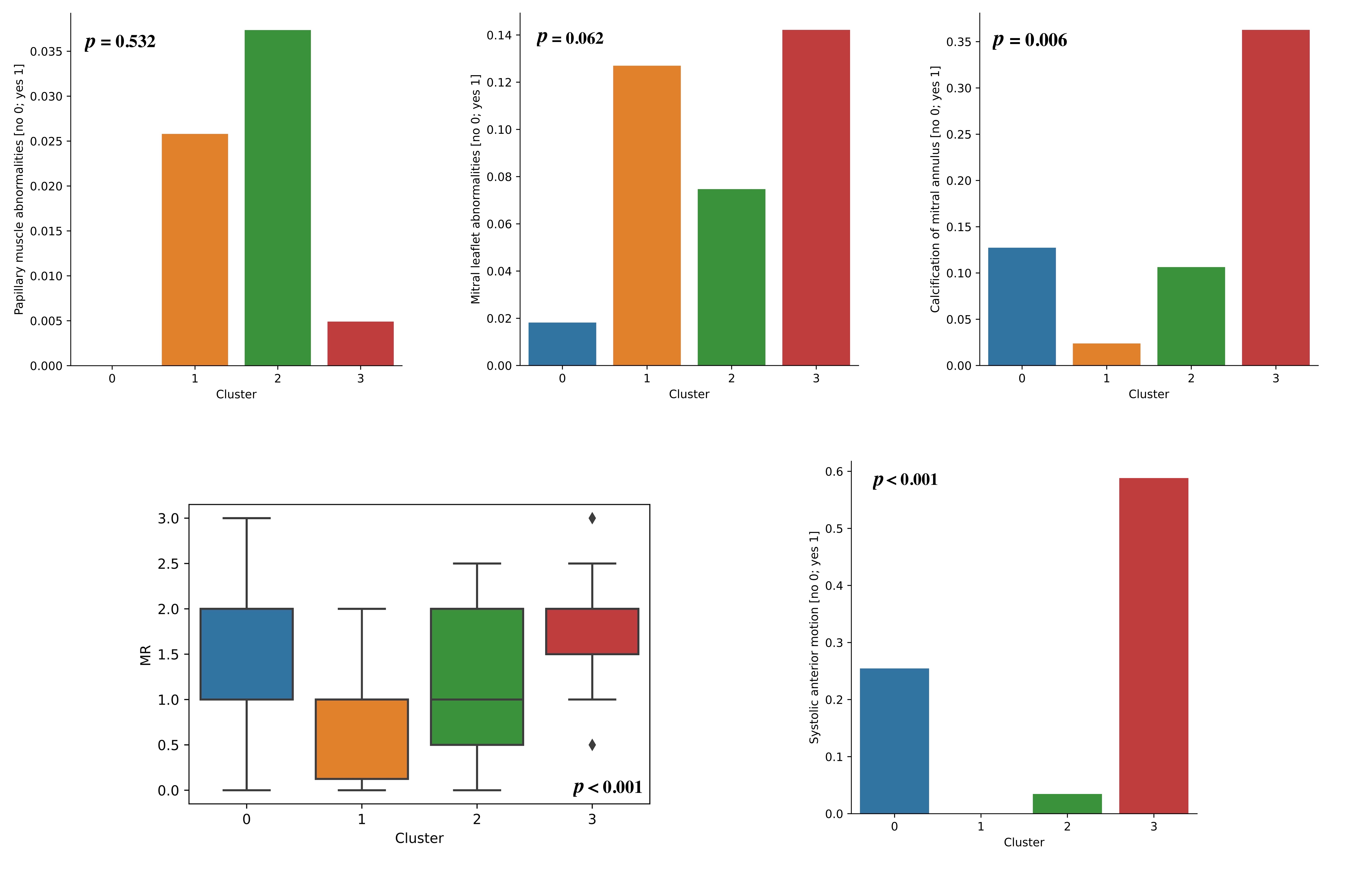

Supplement: Supplementary file 1 [file life-12-01566-s001.zip › Figure S14, Mitral apparatus irregularities, mitral regurgitation, and systolic anterior motion.jpg]

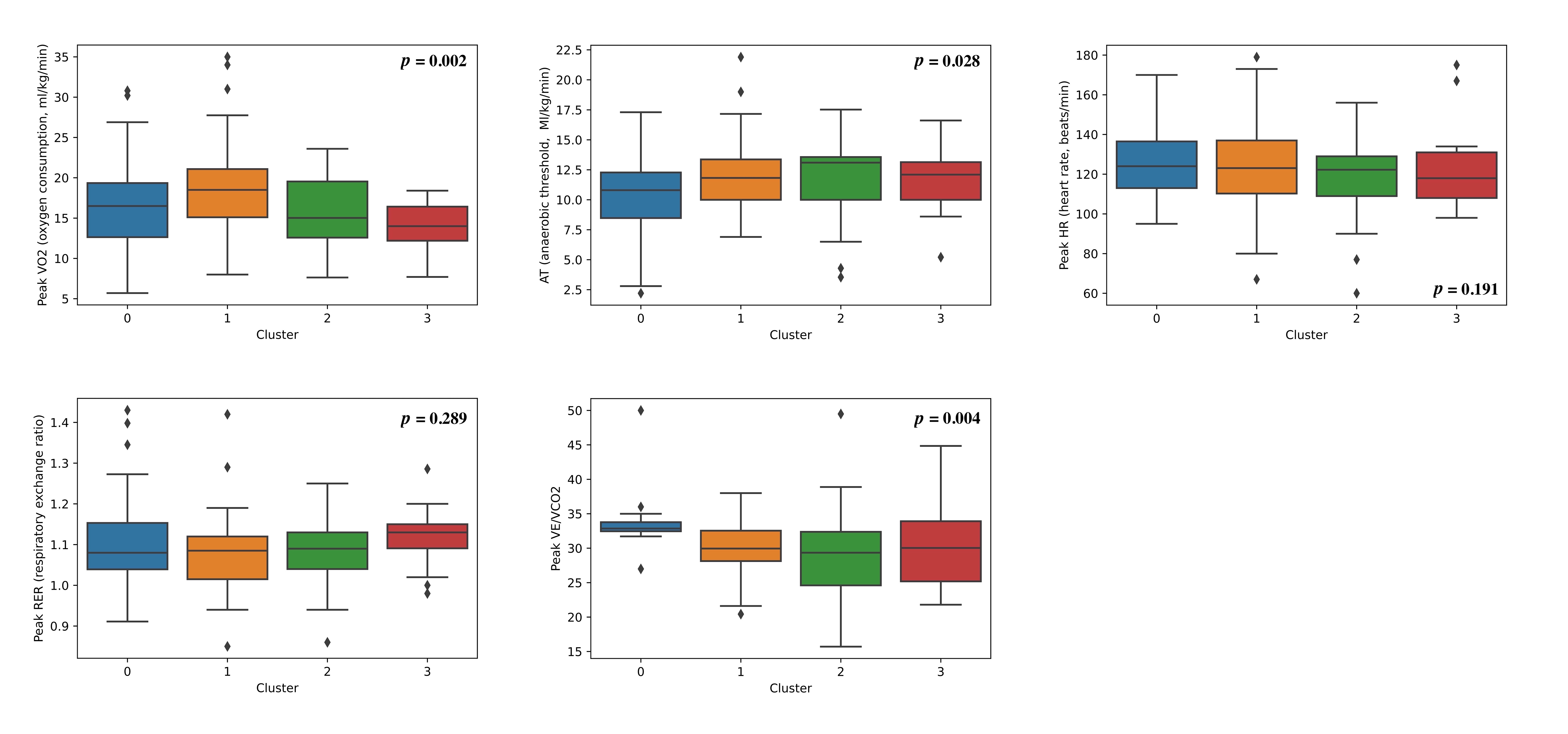

Supplement: Supplementary file 1 [file life-12-01566-s001.zip › Figure S15, CPET.jpg]

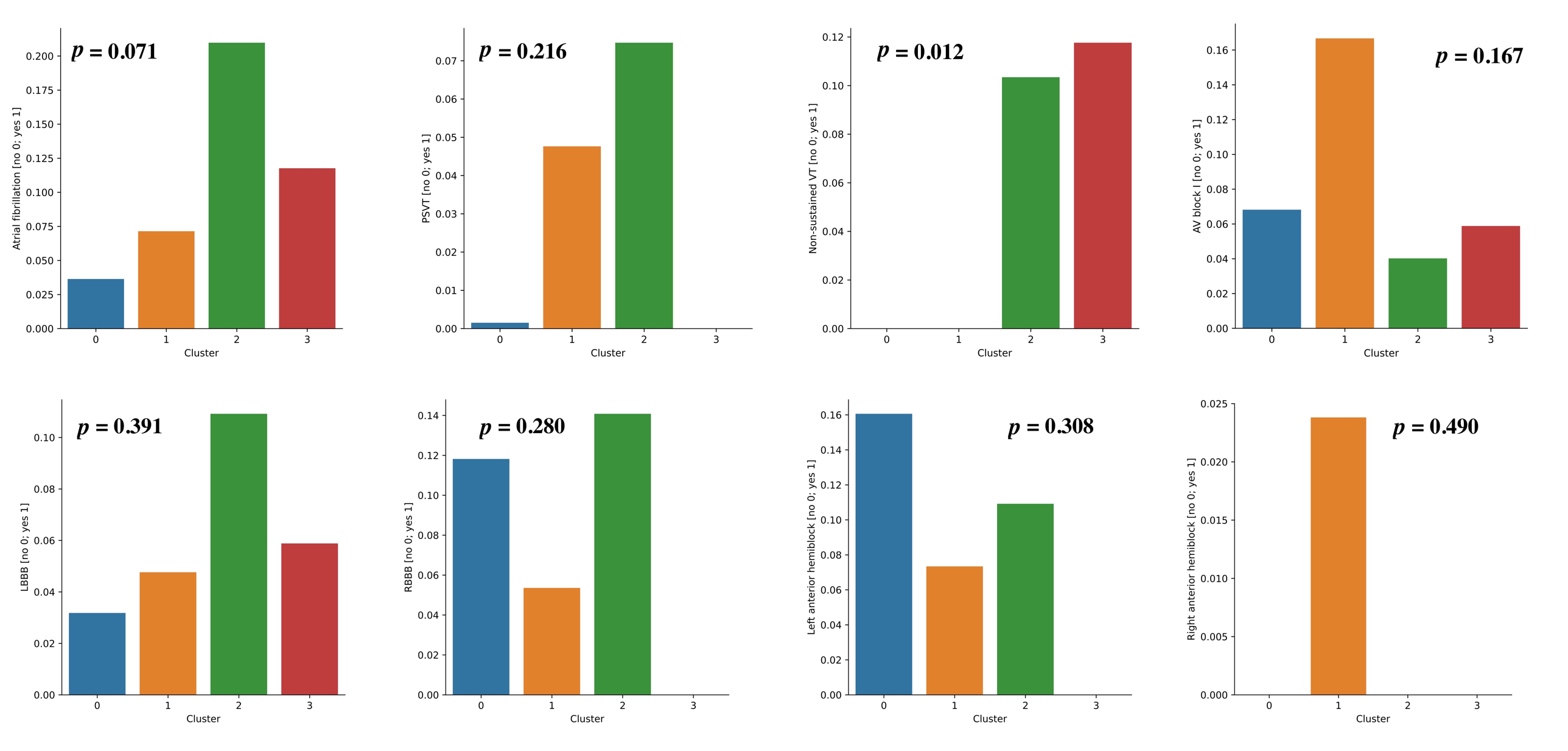

Supplement: Supplementary file 1 [file life-12-01566-s001.zip › Figure S16, Rythm and conduction.jpg]

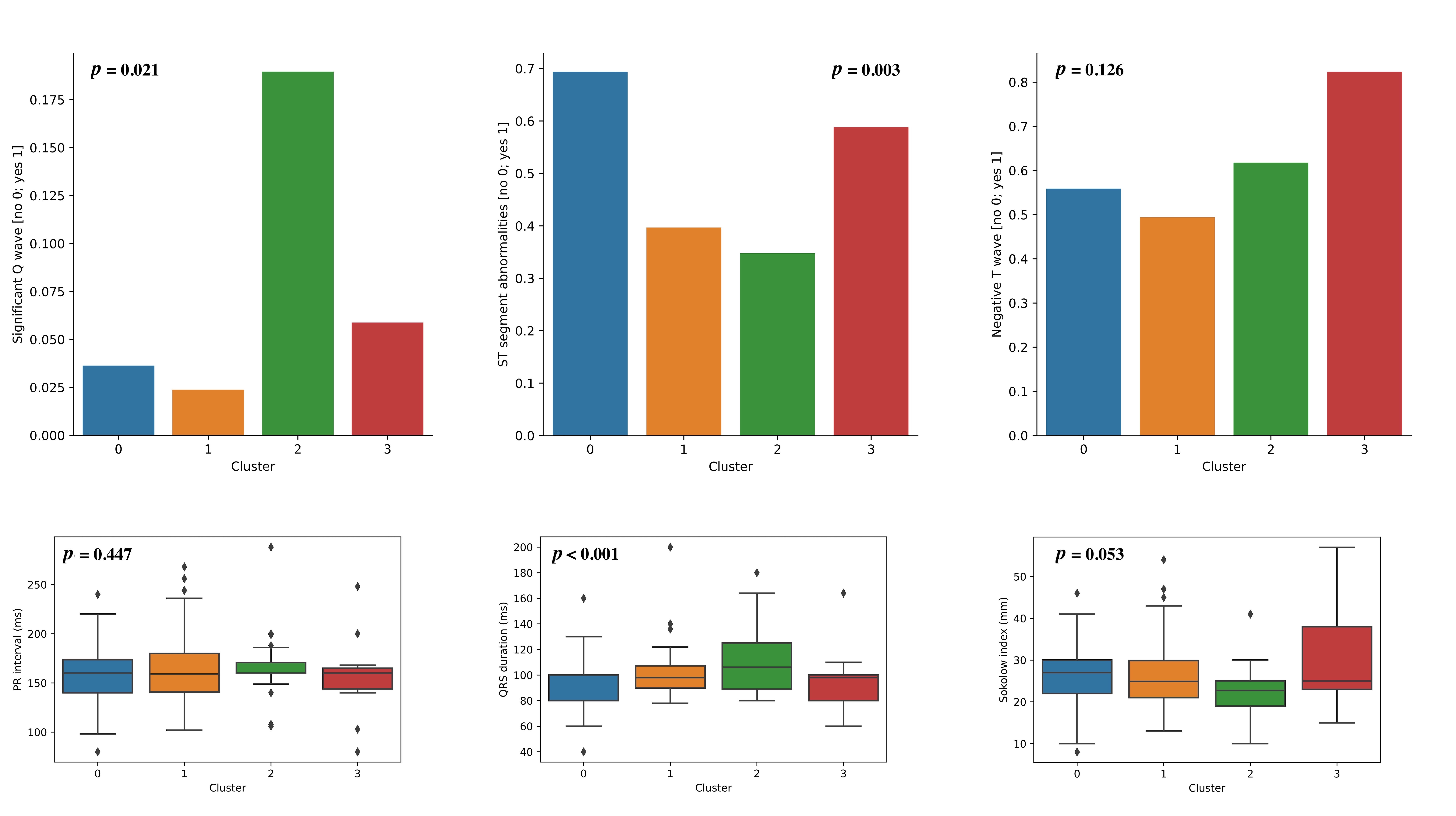

Supplement: Supplementary file 1 [file life-12-01566-s001.zip › Figure S17, Ischemia and Sokolow index.jpg]

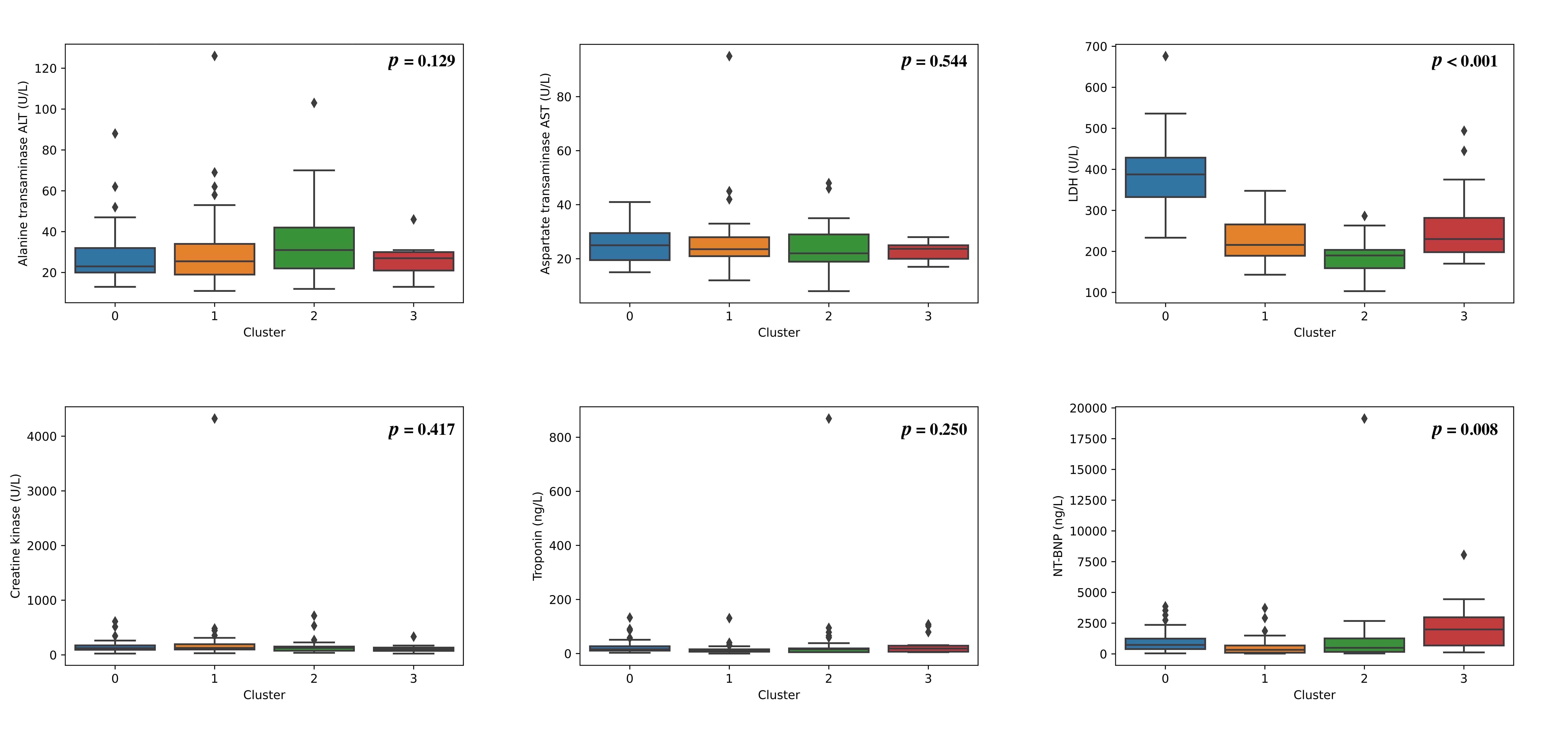

Supplement: Supplementary file 1 [file life-12-01566-s001.zip › Figure S18, Enzymes and cardiac biomarkers.jpg]

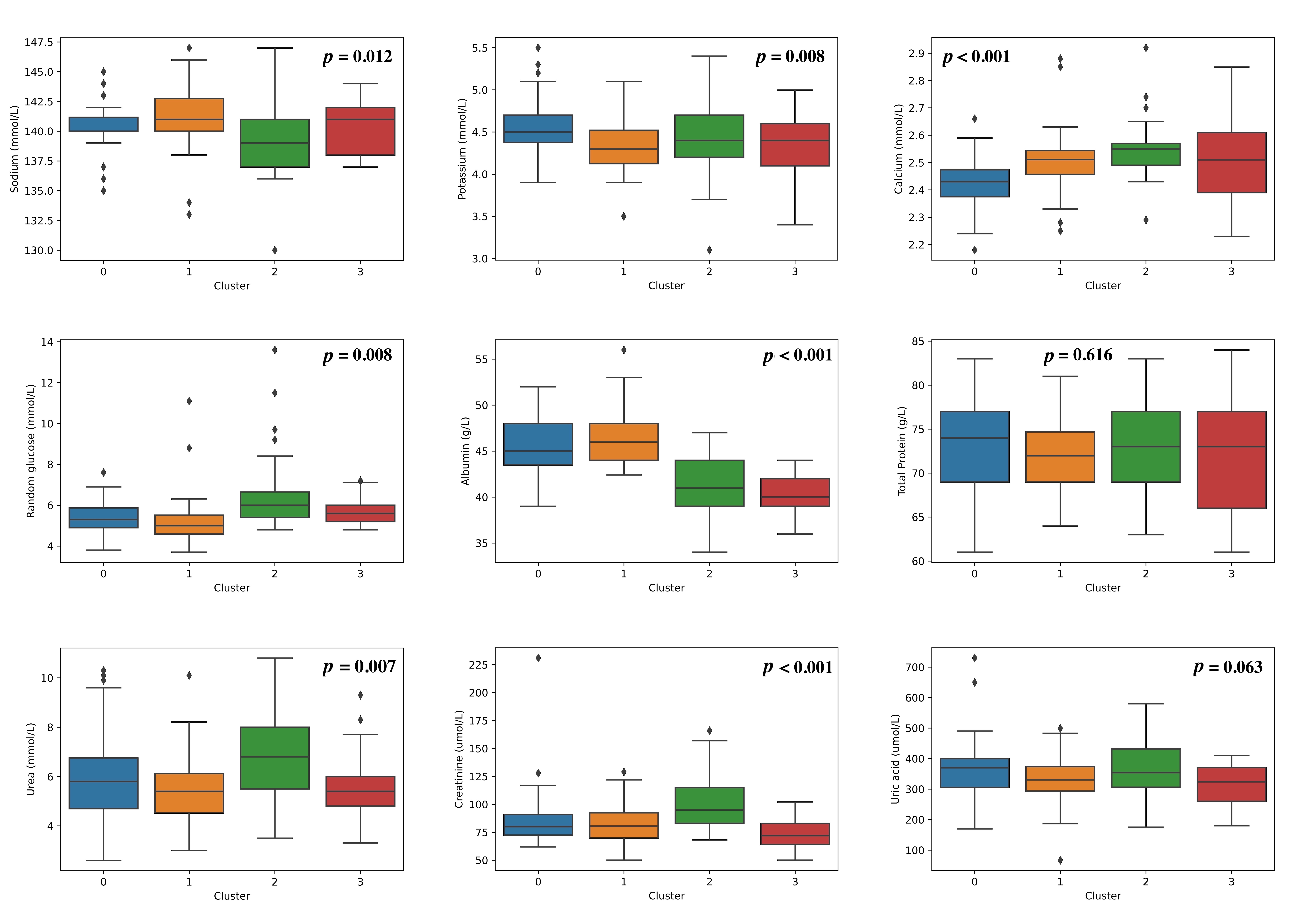

Supplement: Supplementary file 1 [file life-12-01566-s001.zip › Figure S19, General clinical chemistry.jpg]

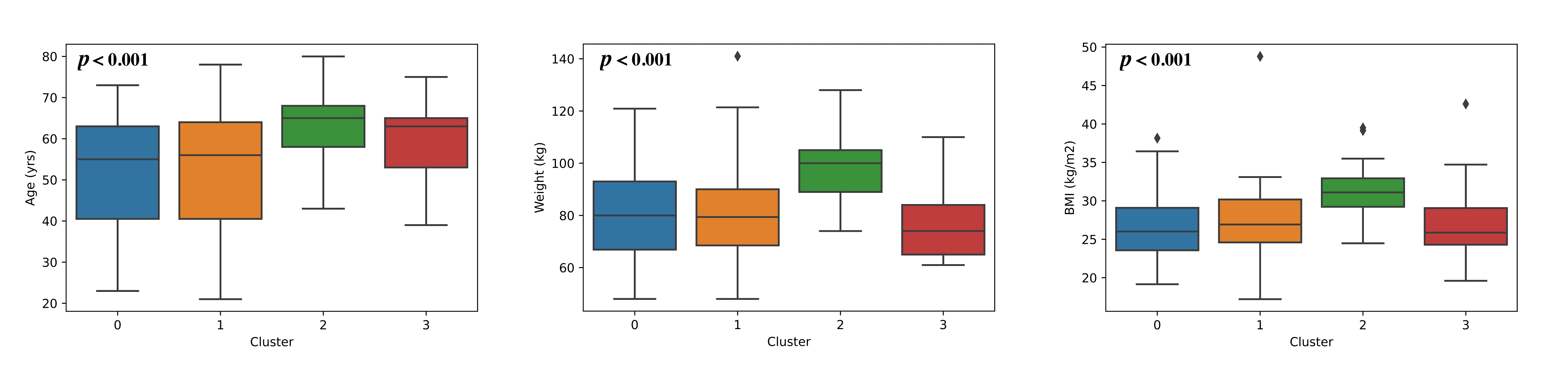

Supplement: Supplementary file 1 [file life-12-01566-s001.zip › Figure S2, Age, weight, and BMI.jpg]

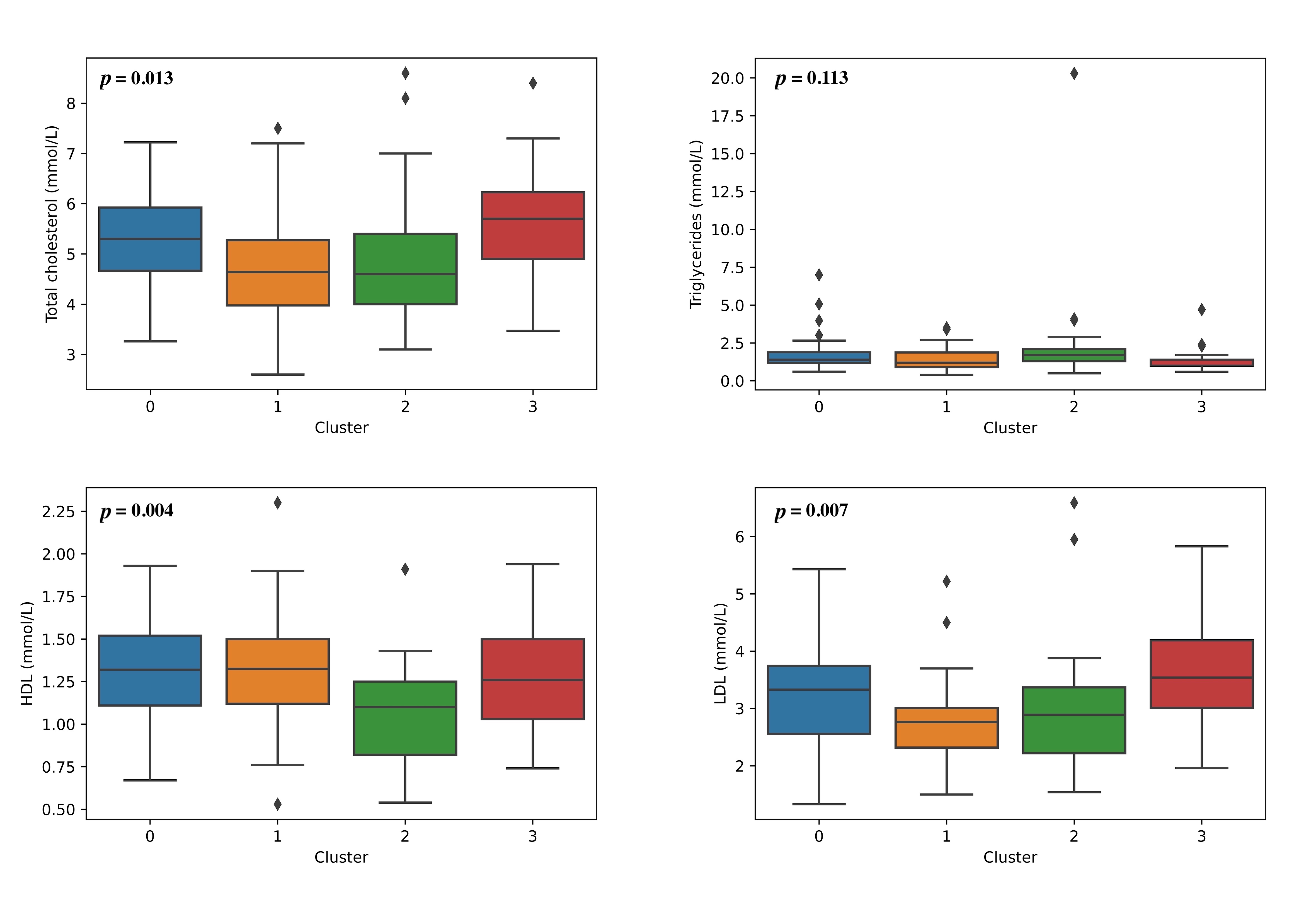

Supplement: Supplementary file 1 [file life-12-01566-s001.zip › Figure S20, Lipid metabolism.jpg]

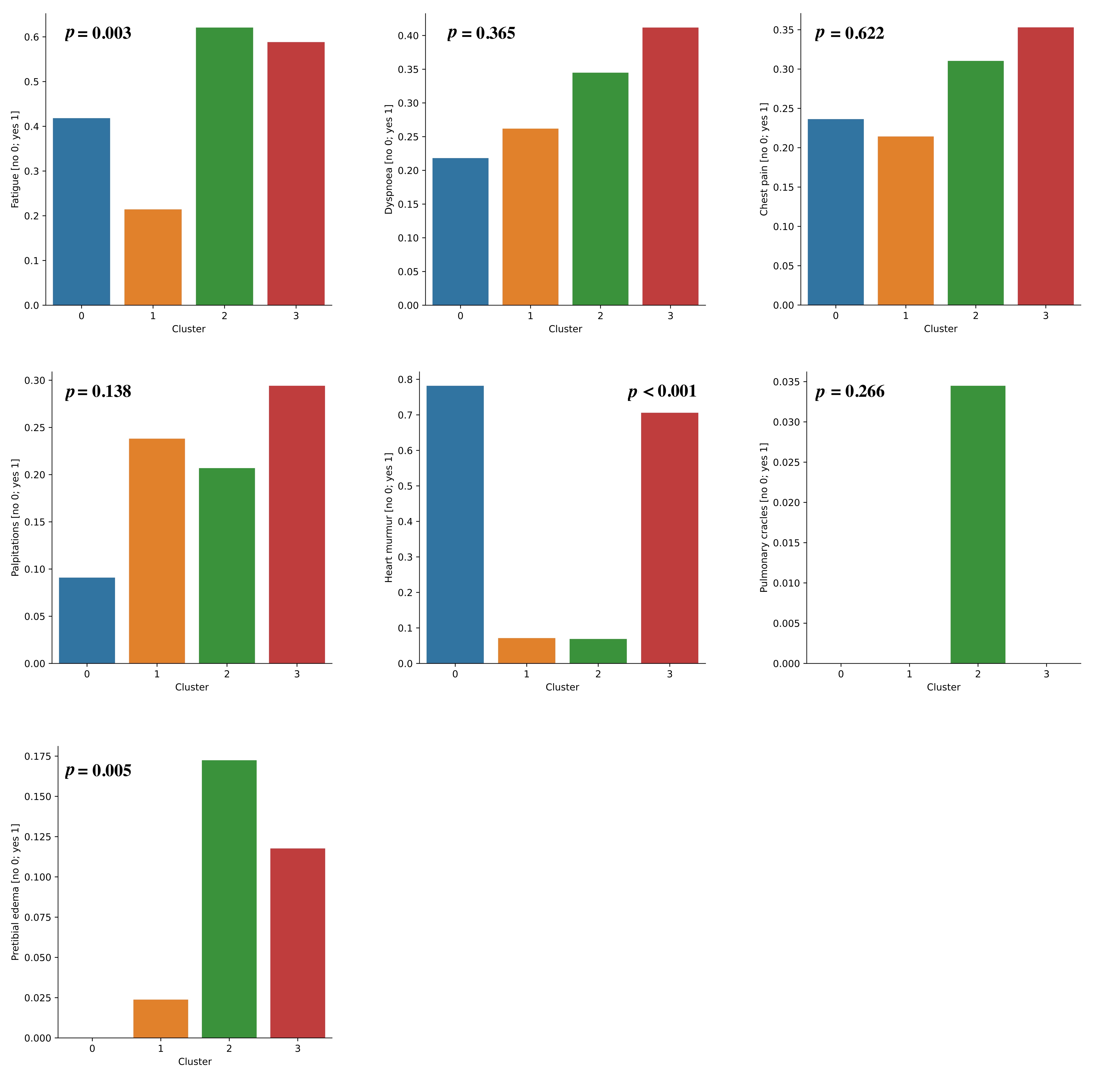

Supplement: Supplementary file 1 [file life-12-01566-s001.zip › Figure S3, Symptoms and signs.jpg]

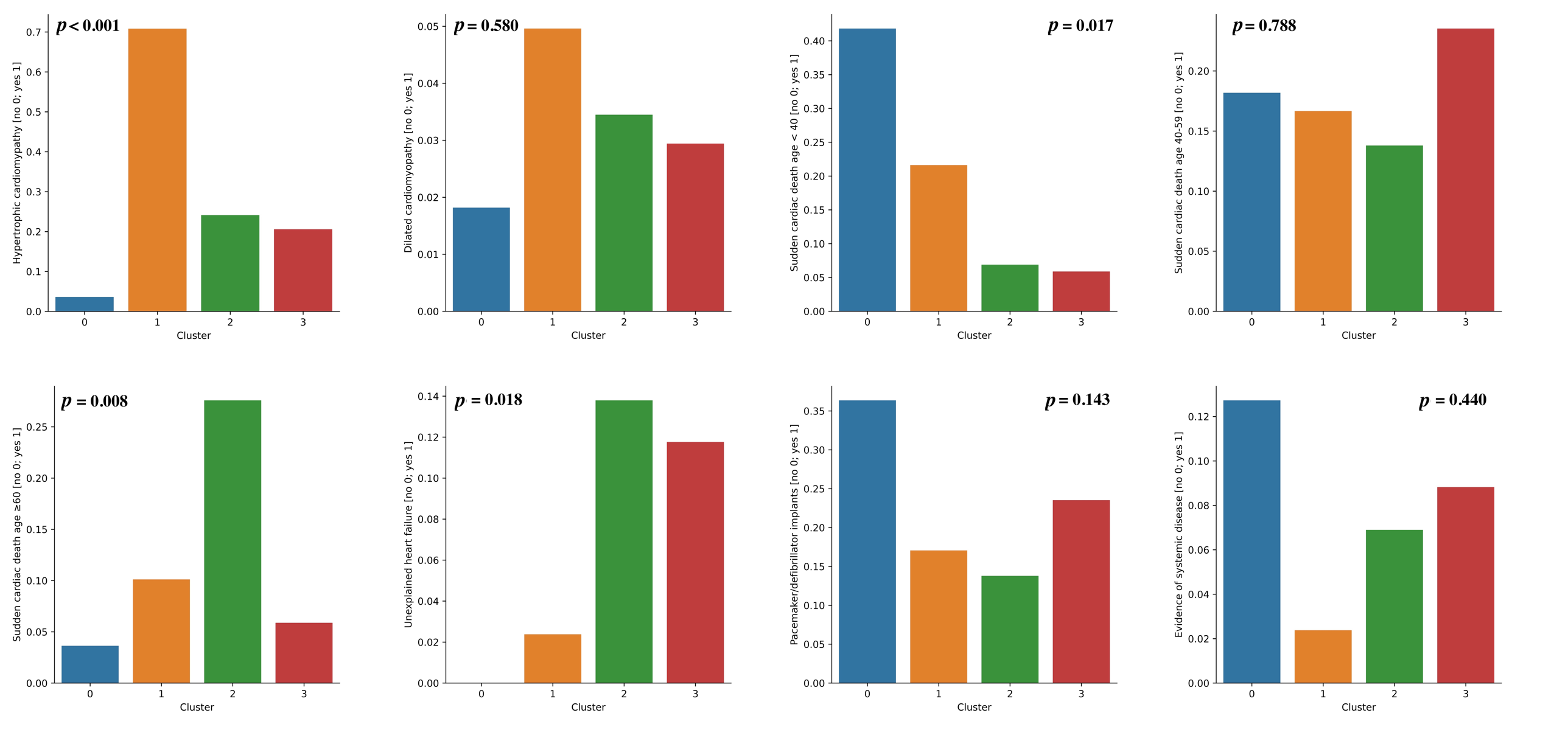

Supplement: Supplementary file 1 [file life-12-01566-s001.zip › Figure S5, Family history.jpg]

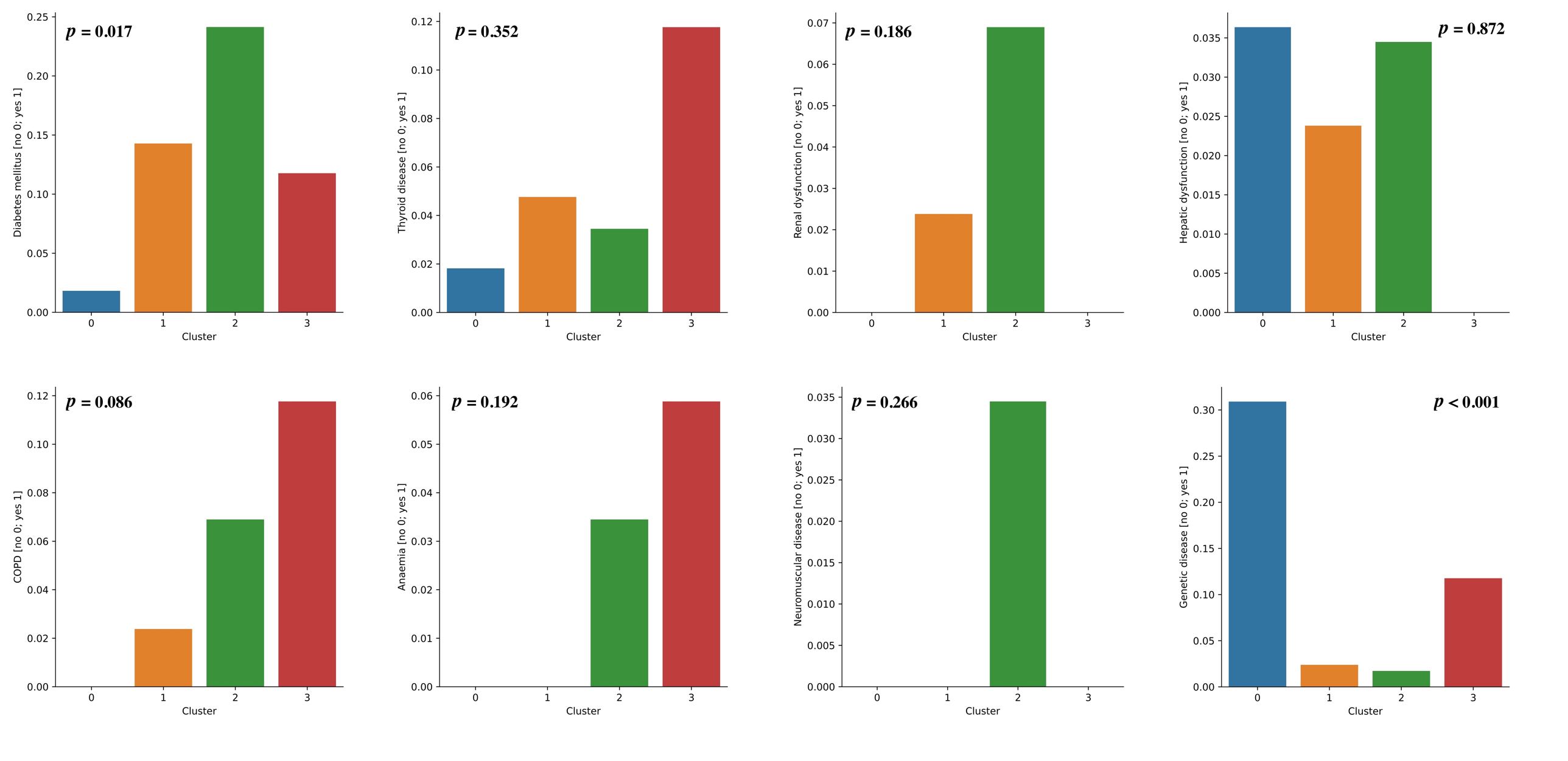

Supplement: Supplementary file 1 [file life-12-01566-s001.zip › Figure S6, Comorbidities .jpg]

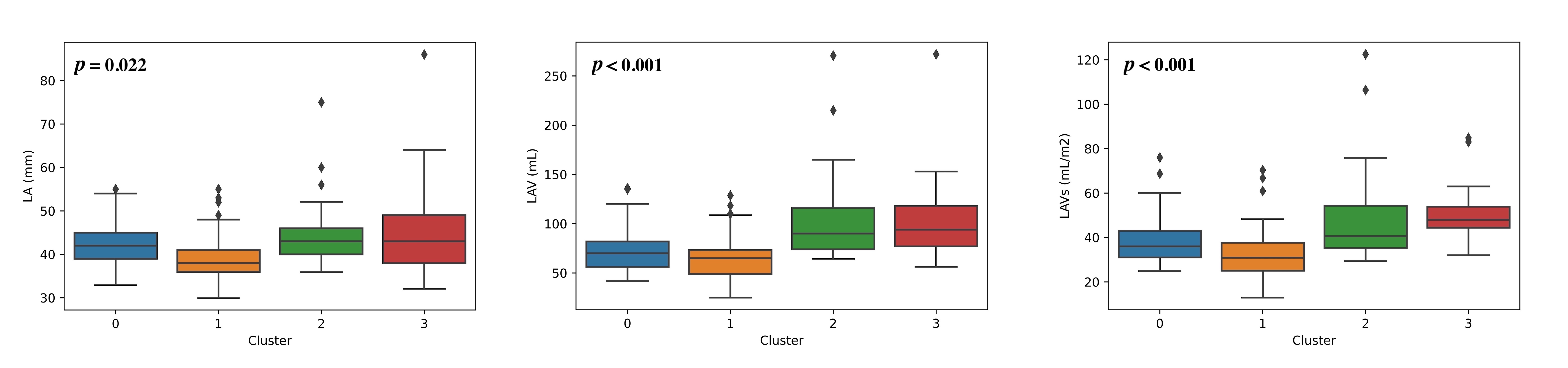

Supplement: Supplementary file 1 [file life-12-01566-s001.zip › Figure S7, Left atrial size.jpg]

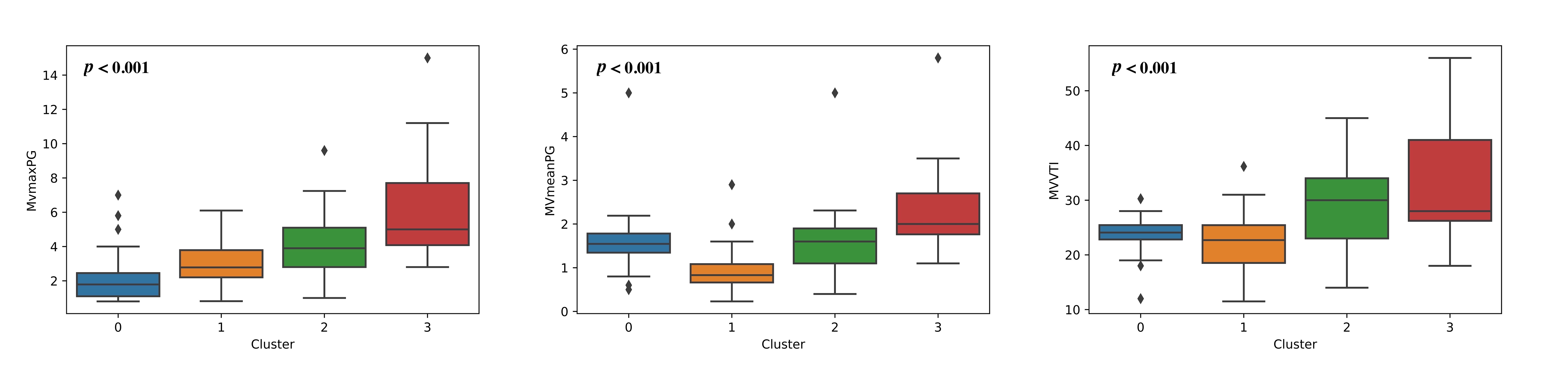

Supplement: Supplementary file 1 [file life-12-01566-s001.zip › Figure S8, Pressure gradients across mitral valve.jpg]

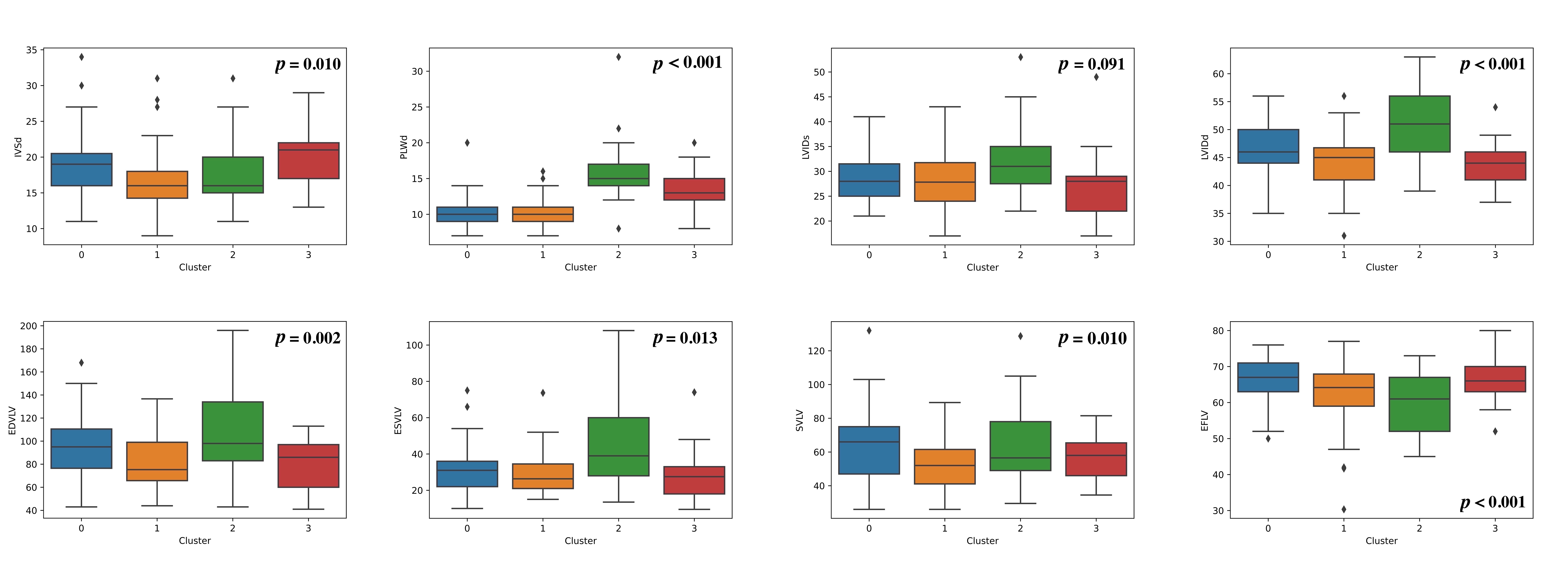

Supplement: Supplementary file 1 [file life-12-01566-s001.zip › Figure S9, Left ventricle.jpg]
